# Integrative analysis of RNA, translation, and protein levels reveals distinct regulatory variation across humans

**DOI:** 10.1101/gr.193342.115

**Published:** 2015-11

**Authors:** Can Cenik, Elif Sarinay Cenik, Gun W. Byeon, Fabian Grubert, Sophie I. Candille, Damek Spacek, Bilal Alsallakh, Hagen Tilgner, Carlos L. Araya, Hua Tang, Emiliano Ricci, Michael P. Snyder

**Affiliations:** 1 Department of Genetics, Stanford University School of Medicine, Stanford, California 94305, USA;; 2 Institute of Software Technology and Interactive Systems, Vienna University of Technology, A-140 Vienna, Austria;; 3 RNA Therapeutics Institute, University of Massachusetts Medical School, Worcester, Massachusetts 01605, USA;; 4 CIRI, International Center for Infectiology Research, Eukaryotic and Viral Translation Team, Université de Lyon, INSERM U1111, Lyon, 69634, France

## Abstract

Elucidating the consequences of genetic differences between humans is essential for understanding phenotypic diversity and personalized medicine. Although variation in RNA levels, transcription factor binding, and chromatin have been explored, little is known about global variation in translation and its genetic determinants. We used ribosome profiling, RNA sequencing, and mass spectrometry to perform an integrated analysis in lymphoblastoid cell lines from a diverse group of individuals. We find significant differences in RNA, translation, and protein levels suggesting diverse mechanisms of personalized gene expression control. Combined analysis of RNA expression and ribosome occupancy improves the identification of individual protein level differences. Finally, we identify genetic differences that specifically modulate ribosome occupancy—many of these differences lie close to start codons and upstream ORFs. Our results reveal a new level of gene expression variation among humans and indicate that genetic variants can cause changes in protein levels through effects on translation.

Deciphering the molecular mechanisms that underlie human variation is essential for understanding human diversity and personalized medicine. To date, genetic variants that affect protein function in humans have been well studied, but those that control protein levels are less well characterized. Yet, misregulation of protein levels can have profound consequences for human health. For example, transcriptional regulatory mutations that increase telomerase gene expression have been identified in ∼70% of melanoma patients (Horn et al. 2013; Huang et al. 2013) and are frequent in several other cancers (Huang et al. 2013). Similarly, changes in protein levels of SHANK3, neuroligins and neurexins have been linked to autism spectrum disorder, schizophrenia, and learning disorders (Darnell 2011). Therefore, understanding how RNA levels and translation efficiency control protein levels on an individual basis is required not only for understanding human phenotypic diversity, but also for personalized medicine as thousands of human genome sequences become available.

Protein expression is determined at many levels, including (1) RNA expression, (2) translation efficiency, and (3) protein stability. Recent studies have begun to unravel the extent of human variation at RNA levels and its control through transcription factor binding sites and chromatin (Stranger et al. 2007; Kasowski et al. 2010, 2013; McDaniell et al. 2010; Montgomery et al. 2010; Pickrell et al. 2010; Pai et al. 2012; Westra et al. 2013). However, protein levels often correlate poorly with RNA expression (Vogel and Marcotte 2012; Ly et al. 2014). Translation efficiency, i.e., the number of proteins synthesized per mRNA, has been suggested to account for a large component of the unexplained variation in protein levels (Schwanhäusser et al. 2011; Marguerat et al. 2012). Although recent studies in yeast have begun to address the genetic control of translation efficiency (Albert et al. 2014; Artieri and Fraser 2014; McManus et al. 2014; Muzzey et al. 2014), little is currently known about variation in translation efficiency and its genetic determinants in humans. Further, an integrated view of how expression is controlled at many different levels is lacking in humans.

Here, we utilized RNA-seq and ribosome profiling to identify ribosome occupancy profiles. Ribosome profiling involves RNase digestion of unprotected RNA and isolation of ribosome-bound mRNA segments. The sequences of ribosome-protected mRNA fragments can then be used to deduce the number of ribosomes per message in conjunction with RNA-seq data. We integrated these measurements with quantitative proteomics to reveal a comprehensive view of the variation in gene expression programs across a diverse set of humans.

## Results

### Measuring ribosome occupancy across individuals at a global scale

**Figure 1.**
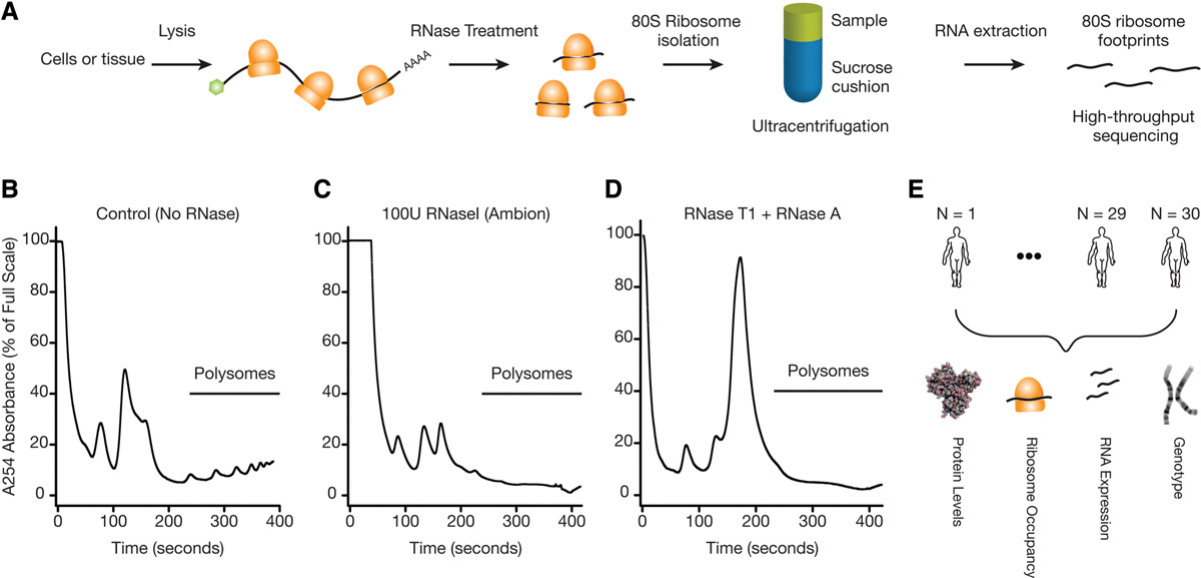
Choice of RNase is critical for generating ribosome profiling data. (*A*) A schematic representation of the ribosome profiling strategy is shown. A key step is the digestion of unprotected RNA segments with an RNase. The ribosome-protected RNA segments are isolated using a sucrose cushion and prepared for high-throughput sequencing. (*B*) Human lymphoblastoid cells (GM12878) were lysed in the presence of cycloheximide. The samples were ultracentrifuged through a 10%–50% sucrose gradient. Samples were fractionated while continuously monitoring absorbance at 254 nm. A representative polysome profile is shown. (*C*) Samples were prepared for ultracentrifugation as in *B* with the following exception: The cleared lysate was incubated with 100 units of RNase I (Ambion) for 30 min at RT before the ultracentrifugation. (*D*) Samples were prepared as in *B*, except 300 units of RNase T1 (Fermentas) and 500 ng of RNase A (Ambion) were used for the RNase digestion step. A complete digestion of polysomes into monosomes was observed. (*E*) Schematic representation of the data sets used in the current study. Genotype, ribosome profiling, RNA-seq, and mass spectrometry-based proteomics data were collected from lymphoblastoid cells derived from a diverse group of 30 individuals.

To measure genome-wide ribosome occupancy of mRNAs, we first improved and adapted the ribosome profiling protocol (Ingolia et al. 2009, 2011, 2012) for lymphoblastoid cell lines (LCLs) (Fig. 1A). A critical step in the ribosome profiling method involves RNase digestion of unprotected RNAs before isolating and sequencing ribosome-associated mRNAs. While optimizing the protocol, we observed that RNase I digestion caused extensive degradation of ribosome integrity (Fig. 1B,C; Supplemental Fig. S1A). The loss of polyribosome signal was not accompanied by a corresponding increase in monosome signal (Fig. 1C; Supplemental Fig. S1A), but rather a shift toward lighter fractions indicative of free and degraded RNAs (Fig. 1C; Supplemental Fig. S1A). Hence, RNase I treatment can lead to a loss in ribosome integrity in addition to producing the expected 80S ribosome footprints (i.e., monosomes).

We tested whether other RNases could alleviate this problem and found that treatment with RNase A and RNase T1 (which collectively cut after C, U, and G) resulted in complete digestion of polyribosomes into monosomes (Fig. 1D; Supplemental Fig. S1A). Recent work in *Drosophila* and other systems also reported the importance of optimizing nuclease digestion to generate robust ribosome profiling data (Dunn et al. 2013; Ricci et al. 2014). Using our optimized ribosome profiling protocol, we generated ribosome occupancy maps for LCLs obtained from 30 individuals of diverse ethnic backgrounds: five Europeans, two Asians, and 23 Yorubans with significant genetic diversity (Fig. 1E). These lines were chosen because (1) their genomes have been sequenced (The International HapMap 3 Consortium 2010; The 1000 Genomes Project Consortium 2012); (2) their relative protein and RNA levels have been previously measured (Khan et al. 2013; Wu et al. 2013); and (3) they can be grown in large quantities. Importantly, the ribosome occupancy maps were based on at least two replicate samples for the majority of individuals. In parallel, we generated 44 deep RNA-seq libraries (with a median of ∼12 million uniquely mapped transcriptome reads) from the same cells and combined these with those from previous work (Pickrell et al. 2010; Lappalainen et al. 2013; ’t Hoen et al. 2013), thereby providing multiple RNA-seq replicates for most individuals.

We leveraged replicate measurements to assess data quality and its dependence on several parameters, including alignment strategy, mRNA enrichment method, PCR artifacts, gene length normalization, and batch effects (Supplemental Fig. S2A–E; Supplemental Methods). In addition to verifying the high quality of the data, replicate measurements also enabled modeling of gene-specific variance in RNA expression and ribosome occupancy per individual, allowing robust derivation of individual-specific translation efficiency estimates. Specifically, we developed a linear modeling–based approach to regress out the effects of RNA expression from ribosome occupancy measurements to calculate translation efficiency (Supplemental Methods).

Finally, for the 28 individuals studied here, we previously measured relative protein abundances via isobaric tag-based quantitative proteomics using the same cell lines (Wu et al. 2013). In total, we present a combined analysis of 133 high-throughput sequencing libraries (83 RNA-seq and 50 ribosome profiling libraries) and extensive protein expression measurements (Fig. 1E).

### Integrative analysis of RNA expression, ribosome occupancy, and protein levels

We first considered the relationship between RNA expression, ribosome occupancy, and protein expression across genes. As expected, RNA expression and ribosome occupancy were highly correlated (Spearman ρ = 0.87, *P*-value < 2.2 × 10^−16^; outlier robust correlation 0.88 using Donoho-Stahel estimator) (Supplemental Fig. S2F), albeit still lower than biological replicates of RNA expression data (Spearman ρ ∼ 0.98, *P*-value < 2.2 × 10^−16^), indicating that control of ribosome occupancy levels is distinct from RNA levels. Importantly, ribosome occupancy correlated better with protein levels than RNA expression correlated with protein levels (Spearman ρ of 0.54 and 0.43, Donoho-Stahel estimator based correlation coefficient 0.56 and 0.42, respectively; permutation test for difference in correlation coefficient *P*-value < 10^−4^) (Supplemental Fig. S2F,G). Consistent with previous results (Ingolia et al. 2009), these results suggest that ribosome occupancy is a better predictor of protein level differences between genes.

**Figure 2.**
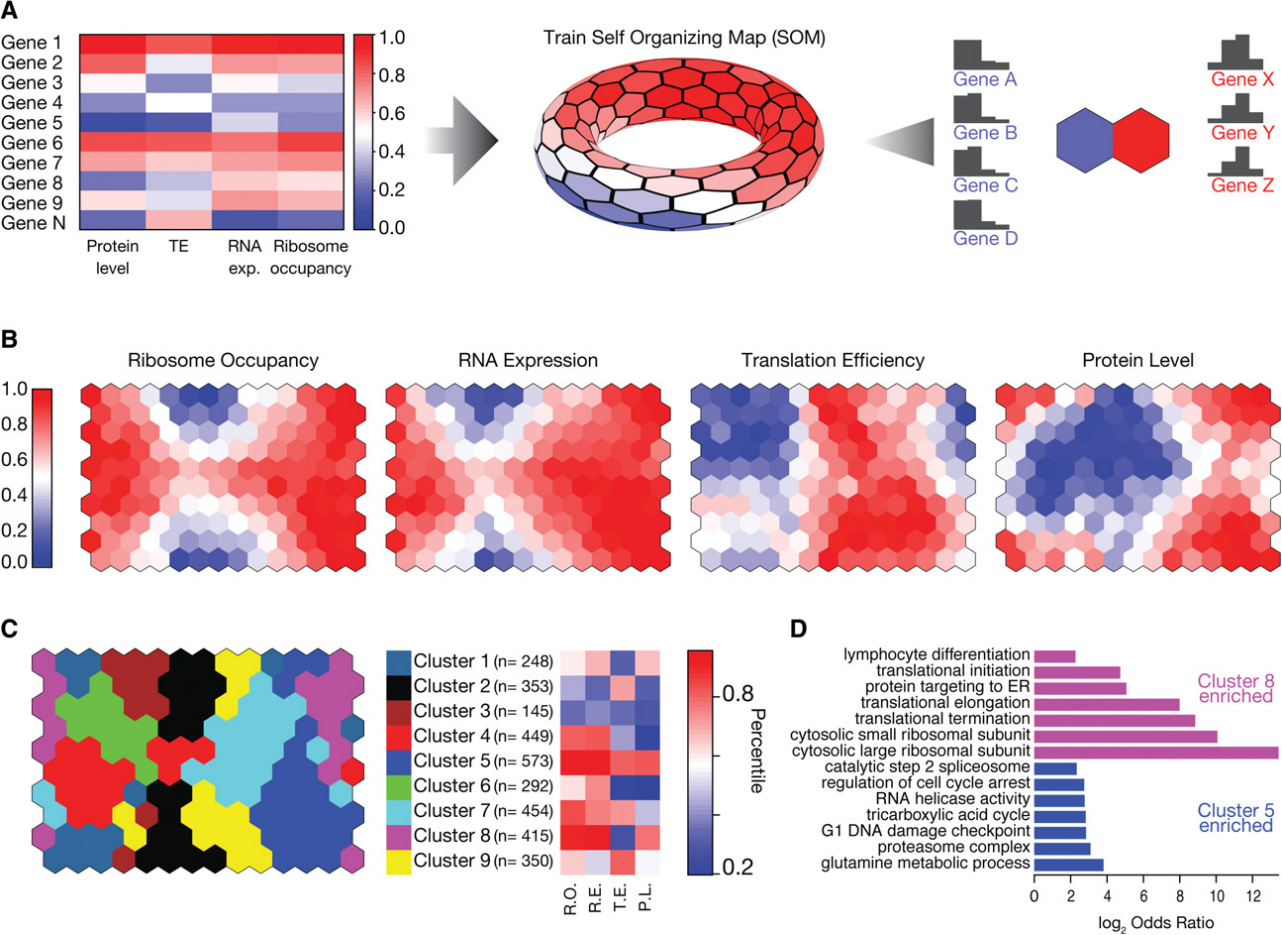
Ribosome occupancy correlates better with absolute protein levels than RNA expression and protein levels. (*A*) A self-organizing map (SOM) was trained using ribosome occupancy, RNA expression, translation efficiency (TE), and protein levels. These measurements were converted into their relative rank order before training. After training, each neuron in the SOM contains several genes sharing similar expression patterns. (*B*) Four different colorings of the trained SOM depict the mean ribosome occupancy, RNA expression, translation efficiency, or protein levels for each neuron. (*C*) Neurons of the SOM were grouped using affinity propagation clustering (Frey and Dueck 2007). Shared coloring between nodes indicates membership to the same cluster. For each cluster, the mean rank in ribosome occupancy (RO), RNA expression (RE), translation efficiency (TE), and protein level (PL) was shown for the representative neuron of the cluster. The number of genes in each cluster (n) is shown. (*D*) For four of nine clusters, significantly enriched gene ontology (GO) terms were identified (FuncAssociate; permutation-based corrected *P*-value < 0.05) (Supplemental Table S1; Berriz et al. 2009). For Clusters 5 and 8, selected GO categories were shown (log_2_ odds ratio). Supplemental Table S1 contains the full list of enriched terms.

Although the correlation analysis reveals pairwise relationships, the interdependencies between RNA expression, translational efficiency, and protein levels are not captured. For example, some genes with high protein levels can have low RNA expression but very high translation efficiency, yielding a decreased correspondence between RNA expression and protein levels. To reveal such interdependencies, we utilized self-organizing maps (SOM), an integrative machine learning method that is robust to noise and allows assessment of all relationships simultaneously (Fig. 2A; Kohonen 1990; Wehrens and Buydens 2007). Because SOMs are sensitive to differences in mean and variance of the input variables, we first converted each measurement into its relative rank order expressed as percentiles ensuring equal weighting of the input variables for the SOM training (Supplemental Methods). After training, each neuron within the SOM contains genes that share a similar pattern of expression and protein level (Fig. 2A; Supplemental Fig. S2H).

The emerging map recapitulated the pairwise relationships between RNA expression, ribosome occupancy, and protein levels across neurons (Fig. 2B). We further grouped neurons in the SOM using a clustering approach (affinity propagation clustering) (Frey and Dueck 2007). This approach uncovered nine clusters in the SOM, revealing the distinct relationships between RNA expression and translation efficiency in determining protein levels. For example, genes in Cluster 6 have relatively high RNA expression but do not reach high protein levels as they are translationally repressed (Fig. 2C).

We then examined functional (GO term) enrichments (Berriz et al. 2003, 2009) across the different clusters within the SOM and found specific functional enrichments for four of the nine clusters (Fig. 2C; Supplemental Table S1). Genes with high translation efficiency and high protein levels were enriched for diverse functional categories such as the proteasome complex, glycolysis, mRNA splicing, and DNA damage checkpoint (*P*-value < 0.05 for all categories using permutation-based multiple hypothesis correction) (Supplemental Table S1; selected examples are shown in Fig. 2D). Conversely, genes associated with translation and cytosolic ribosome constituents were enriched among those that exhibited very high RNA and protein levels despite having lower translation efficiencies (*P*-value < 0.05 for all categories using permutation-based multiple hypothesis correction) (Supplemental Table S1; Fig. 2D), raising the possibility that higher protein stability or feedback mechanisms on translation efficiency modulate the levels of translation components. These findings indicate that some sets of functionally coherent genes adopt alternative strategies to achieve their respective steady-state protein levels.

### Gene expression variability between individuals

**Figure 3.**
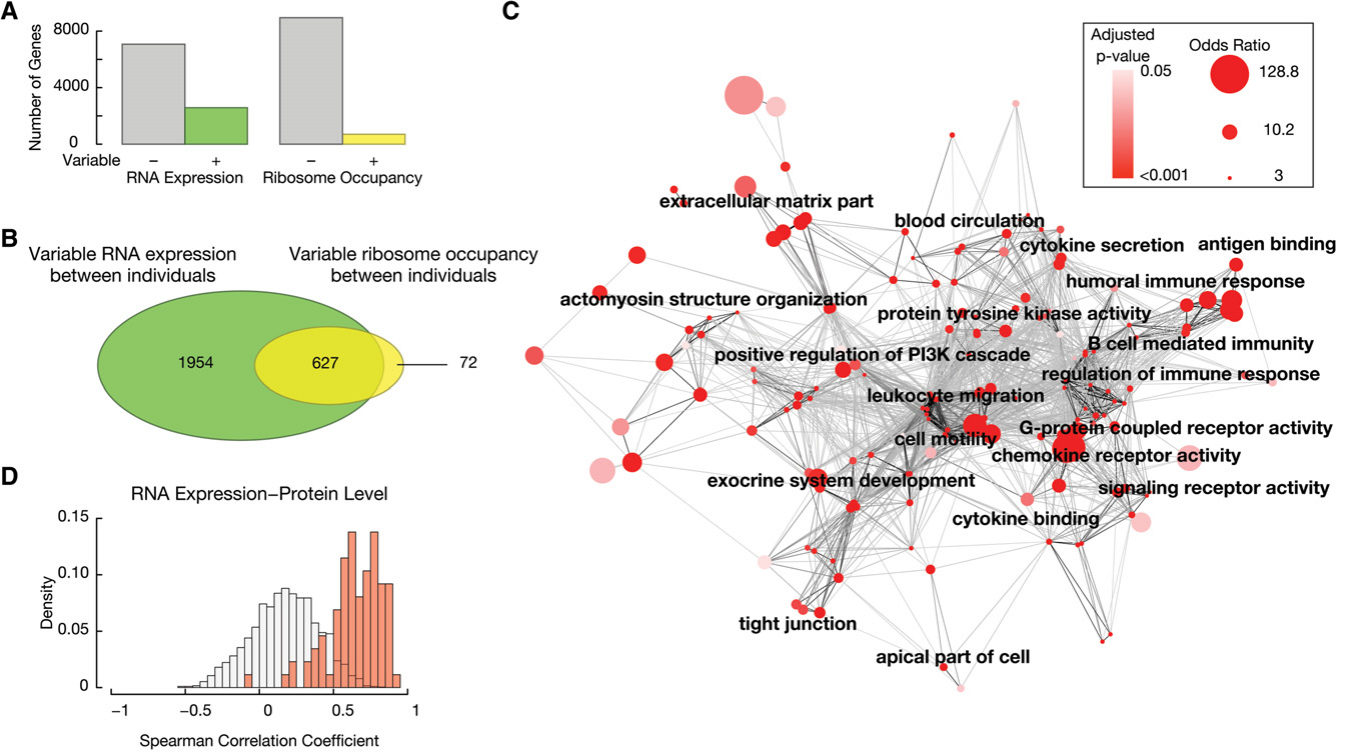
Identification of genes with significant inter-individual variability in RNA expression and ribosome occupancy improves the ability to identify personal differences in protein levels. (*A*) Ribosome occupancy and RNA expression was modeled using a linear mixed model treating individuals as a random effect and mean expression as the fixed effect. A simulation-based exact likelihood ratio test (Scheipl et al. 2008) was used to compare the linear mixed model to a linear model that did not include the individual as a predictor. The number of genes that show significant inter-individual in RNA expression or inter-individual variation in ribosome occupancy is plotted (Holm's corrected *P*-value < 0.05). (*B*) The Venn diagram depicts the overlap between the two groups. (*C*) Enriched gene ontology (GO) terms among genes with significant inter-individual variation in both RNA expression and ribosome occupancy was determined using FuncAssociate (Berriz et al. 2009). Cytoscape (Smoot et al. 2011) was used to visualize the enriched GO terms (permutation test corrected *P*-value < 0.05, odds ratio > 3) (Supplemental Table S2). Nodes correspond to GO terms and are colored by the corrected *P*-value. The size of the node is proportional to the logarithm of the odds ratio. The similarity between GO terms was quantified using Kappa similarity. The strength of the similarity was visualized using darker edge colors (Supplemental Methods). An edge-weighted spring embedded layout is shown. (*D*) For each gene, Spearman correlation was calculated between individual specific RNA expression and relative protein levels. The distribution of the correlation coefficients was plotted as a density. Genes that showed significant variation in both RNA expression and ribosome occupancy between individuals are plotted with red bars and genes without detectable variation in RNA expression and ribosome occupancy are shown with white bars.

We next focused on how ribosome occupancy and RNA expression differ between individuals. We leveraged replicate measurements and identified genes with significant inter-individual variance in RNA expression or ribosome occupancy, exceeding technical noise. We found that ∼27% of genes had statistically significant inter-individual variation in RNA expression compared to only ∼7% of genes that had detectable variation in ribosome occupancy (Holm’s method adjusted *P*-value < 0.05 based on a simulation based likelihood ratio test) (Fig. 3A,B; Holm 1979). Consequently, ∼20% of all genes exhibit inter-individual RNA expression variation that is not reflected in ribosome occupancy. These results were not explained by different sensitivities of the measurements (Supplemental Fig. S3A). These results were also consistent when restricting the analysis to only the Yoruban individuals or when excluding RNA expression data not generated by our laboratory, indicating the robustness of the results.

Genes that exhibited significant inter-individual variation in both RNA expression and ribosome occupancy were highly enriched for gene ontology terms, including “chemokine receptor activity,” “complement activation,” “leukocyte migration,” and “antigen binding” (*P* < 0.05 permutation-based multiple hypothesis correction) (Fig. 3C; Supplemental Table S2), indicating a role in immune functions. Consistently, protein levels that exhibit the most variation between individuals were previously shown to be enriched for “immune system process” (Wu et al. 2013). These functional categories are highly specific to the function of the studied cell type, LCLs (Fig. 3C; Supplemental Table S2). Given that genes with significant inter-individual variation were directly pertinent to the function of the cell line studied here, it is likely that carrying out similar studies in other cell types will expand the set of genes whose expression levels differ significantly between individuals.

Within genes that exhibited significant inter-individual variation in both RNA expression and ribosome occupancy, we identified three subsets. Within the first subset, the variability in RNA expression was comparable to variability in ribosome occupancy (Supplemental Fig. S3E). This first subset contained 54% of all genes exhibiting inter-individual variation in both RNA expression and ribosome occupancy. The second subset consisted of genes that had higher RNA-level variability compared to ribosome occupancy variability. This subset encompassed nearly twice as many genes as the third subset, where ribosome occupancy variability was higher than that of RNA expression (Supplemental Fig. S3E). These results were consistent with the observation that for many genes, inter-individual RNA expression variation is not reflected in ribosome occupancy. Taken together, our results are consistent with yeast studies that reported translational buffering of divergent RNA expression (Artieri and Fraser 2014; McManus et al. 2014). However, we note that an alternative explanation of our findings is the presence of an untranslated pool of mRNA (e.g., nuclear-retained or sequestered cytoplasmically in P bodies) that is variable between individuals.

A small, but interesting fraction of genes (0.7%) exhibited differential ribosome occupancy between individuals with no apparent differences at the RNA level (Supplemental Table S3). These were enriched in genes coding for proteins involved in “cellular response to chemical stimulus” and the “Golgi apparatus” (*P* < 0.05 permutation-based multiple hypothesis correction) (Supplemental Table S2). These results suggest that translational control may play important roles in cellular signaling, whereby rapid cellular responses are often required.

### Relationship between individual differences in protein levels, ribosome occupancy, and RNA expression

An outstanding question in understanding phenotypic variation is how individual-specific protein levels relate to corresponding differences in gene expression. We previously measured relative protein levels for approximately 6000 proteins using the same cell lines (Wu et al. 2013). As expected, the protein level measurements were skewed toward genes that are more highly expressed and translated (Wilcoxon rank-sum test *P* < 2.2 × 10^−16^) (Supplemental Fig. S3B). We first calculated the correlation between RNA expression and the corresponding protein level across individuals. Consistent with previous results (Wu et al. 2013), the median correlation coefficient was 0.22, with 11% of genes showing a statistically significant correlation (Spearman correlation coefficient, 5% FDR using the Benjamini-Hochberg method) (Fig. 3D; Supplemental Fig. S3C; Benjamini and Hochberg 1995).

We next repeated this analysis for the set of genes that we identified as having significant RNA expression variability between individuals. Among this subset, relative protein levels and RNA expression had a median correlation coefficient of 0.43 (Spearman correlation coefficient) (Supplemental Fig. S3D), indicating a partial correlation between RNA and protein variability.

Finally, we tested whether joint measurement of RNA expression and ribosome occupancy improved this correspondence. Specifically, we considered genes that exhibit significant inter-individual variation in both ribosome occupancy and RNA expression. Strikingly, 83% of these genes had statistically significant correlation between differences in protein levels and RNA expression with a median correlation coefficient of 0.67 (Spearman correlation coefficient, 5% FDR using Benjamini Hochberg method) (Fig. 3D; Supplemental Fig. S3C). The large difference between the correlation coefficients indicates that measuring both ribosome occupancy and RNA levels simultaneously greatly improves the ability to identify gene expression variability between individuals that will eventually result in personal differences in protein levels.

### Genetic determinants of variability in ribosome occupancy

We next investigated whether genetic differences between individuals were associated with the observed variation in gene expression, specifically at the ribosome occupancy level. We used two complementary approaches. First, we used the 21 unrelated individuals from the Yoruban population and conducted a *cis*-quantitative trait loci (*cis*-QTL) mapping approach. Using the *cis*-QTL mapping strategy, we identified significant association between single nucleotide polymorphisms (SNPs) and ribosome occupancy for 67 genes (30% FDR) (Supplemental Fig. S4A–D; Supplemental Table S4). Although 34 of the 67 ribosome occupancy QTLs (roQTLs) were not associated with significant differences in RNA expression (nominal association *P*-value > 0.05), this analysis cannot conclusively show that these roQTLs are not associated with RNA expression. Overall roQTLs had consistent effects on RNA expression and protein levels (Spearman ρ = 0.86, *P* < 2.2 × 10^−16^) (Supplemental Fig. S4A–D). Consistent with recent work comparing two yeast strains (Albert et al. 2014), these results suggest that genetic effects that were propagated through RNA expression to ribosome occupancy caused consistent changes in protein levels for this set of genes.

### The role of uORFs in modulating ribosome occupancy

We next adopted a targeted approach that was both better powered and enabled detection of combined effects of multiple genetic variants on ribosome occupancy. We first analyzed variants modifying upstream open reading frames (uORFs), which can alter protein expression by regulating translation (Wethmar et al. 2014). In humans, approximately half of annotated transcripts contain uORFs, and presence of uORFs is widely polymorphic across individuals (Supplemental Table S5; Calvo et al. 2009; Barbosa et al. 2013; Waern and Snyder 2013). Disruption of a uORF in the *HR* gene has been previously shown to lead to Marie Unna hereditary hypotrichosis by modulating the translation of the main ORF, suggesting that human disease can be associated with changes in uORFs (Wen et al. 2009).

**Figure 4.**
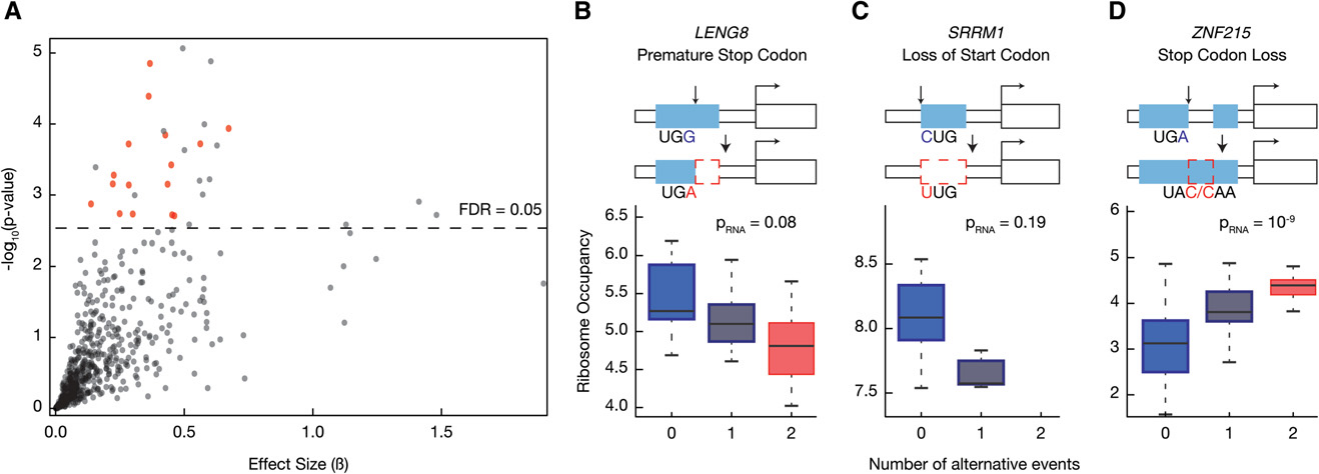
Nucleotide variants that modify upstream ORFs can alter ribosome occupancy of the main coding region. (*A*) We identified single nucleotide polymorphisms that generate, delete, or otherwise modify an upstream open reading frame (uORF). We tested whether changes to uORFs affected ribosome occupancy of the main coding region using a linear regression framework. The absolute value of the effect size from the regression was plotted against the *P*-value of association. For 17 uORF changes shown with red circles, the association was solely with ribosome occupancy (nominal *P*-value > 0.05 or opposite signed regression coefficients for RNA expression). Supplemental Table S5 shows the robustness to population stratification and linear mixed model. (*B*) A SNP in the 5′ UTR of the *LENG8* gene introduces a premature in-frame stop codon that shortens an existing uORF. This event results in lower ribosome occupancy of the main coding region, as shown in the boxplot (*p*_Ribo_ = 0.002). The horizontal bar reflects the median of the distribution, and the box depicts the interquartile range. The whiskers are drawn to 1.5 times the interquartile range. (*C*) In another example, *SRRM1*, a SNP completely eliminates an existing uORF by removing its start codon. The loss of this uORF is associated with reduced ribosome occupancy of the main coding region (*p*_Ribo_ = 0.0004; *p*_RNA_ = 0.19). (*D*) The reference sequence of *ZNF215* gene has two short uORFs. Two different genetic variants eliminate the stop codon of the first uORF (UGA to UAC or UGA to CAA), resulting in merging of the two short uORFs into a single long uORF. The merging of the uORF significantly modulates both ribosome occupancy and RNA expression (*p*_Ribo_ = 0.0001 and *p*_RNA_ = 10^−9^, respectively).

We correlated genetic alterations to uORF presence with ribosome occupancy of the main coding region and found 33 transcripts with significant association (5% FDR) (Fig. 4A–D; Supplemental Fig. S4E,F). One particular advantage of this targeted approach was the ability to detect changes to uORFs caused by multiple genetic variants. For example, two different SNPs in the *ZNF215* gene result in merging of two uORFs by removing a stop codon (Fig. 4D). Merging of the uORFs significantly increased ribosome occupancy of the main coding region (*P* < 0.001) (Fig. 4D).

In addition to impacting translational efficiency, nucleotide variants changing uORFs may alter RNA abundance. For example, they may change transcript stability by triggering nonsense-mediated decay (Kervestin and Jacobson 2012). Alternatively, the variant or variants in linkage disequilibrium may alter transcriptional output as a proximal element downstream from the transcription start site. Of the 33 significant associations between changes in uORFs and ribosome occupancy, ∼52% (17 of 33) also had a significant effect on RNA levels (nominal *P*-value < 0.05), indicating the presence of uORFs and RNA levels are often coupled. However, for 16 other genes, the observed effect was solely on ribosome occupancy, suggesting direct modulation of the translation efficiency of the main reading frame (Fig. 4A,B, Supplemental Table S5). We further observed that presence of uORFs could be associated with both increased and decreased ribosome occupancy of the main coding region (Supplemental Fig. S4G). We verified the robustness of these results by limiting the analysis to data from Yoruban individuals and using an alternative statistical framework based on linear mixed models (Supplemental Fig. S4E,F; Supplemental Table S5). These results reveal that natural genetic variation within the human population can specifically cause personal differences in translation through changes to uORFs.

### The role of sequences surrounding the start codon in modulating ribosome occupancy

**Figure 5.**
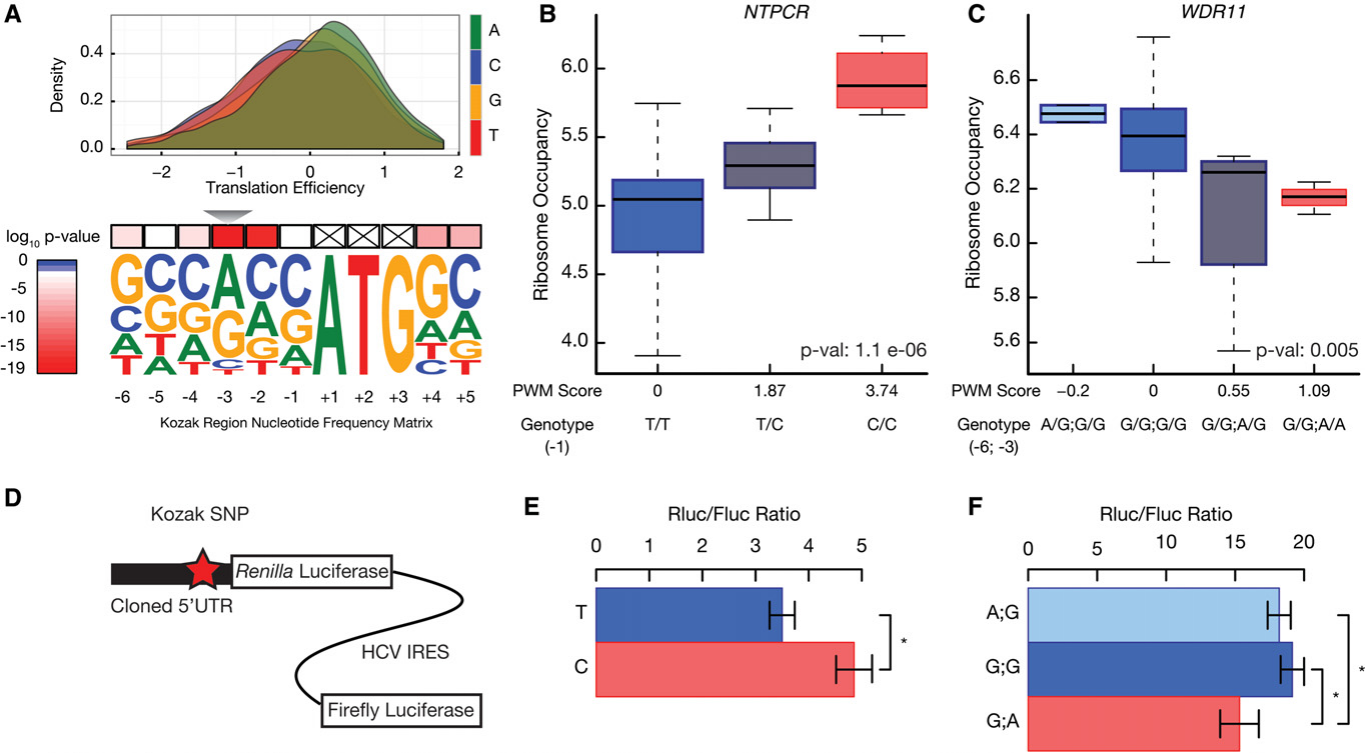
Nucleotide variants modulating the sequence around the translation initiation site alter translation efficiency. (*A*) The Kozak region is defined as the 6 nt preceding and 2 nt following the start codon. The derived position weight matrix was visualized using WebLogo (Crooks et al. 2004). The *upper* panel shows the effects of each nucleotide at the −3 position on translation efficiency. The effect of nucleotides on translation efficiency was tested using the Kruskal–Wallis test. (*B*) The effect of a Kozak region variant on the ribosome occupancy of *NTPCR* was assessed using a linear model (*P*-value = 1.1 × 10^−6^). A boxplot was used to visualize the distribution of ribosome occupancy for individuals with given genotypes. The horizontal bar reflects the median of the distribution and the box is drawn to depict the interquartile range. (*C*) *WDR11* had two naturally occurring SNPs in its Kozak region. An additive model was adopted to calculate the change in the position weight matrix score of the Kozak region. (*D*) 5′ UTRs with or without Kozak variants were cloned into a translation efficiency reporter. The reporter expresses a biscistronic mRNA, in which the *Renilla* luciferase is translated under the control of the cloned 5′ UTR, and the firefly luciferase is translated under the control of Hepatitis C virus (HCV) internal ribosome entry site (IRES). (*E*,*F*) The ratio of *Renilla* to firefly luciferase activity was plotted for *NTPCR* (*E*) and *WDR11* (*F*). Error bars represent SEM. The difference between the ratios was assessed using a two-sided two-sample *t*-test. (*) *P*-value < 0.05.

We next analyzed the Kozak sequence, the region surrounding the start codon for its effect on translation efficiency (Fig. 5A). Previous work has suggested that Kozak sequence is important for both start codon selection and translation efficiency of specific transcripts (Kozak 1987). However, the extent and impact of natural genetic variation affecting the Kozak sequence and the global effect of the Kozak region on translation efficiency have not been studied.

We first determined whether certain positions of the Kozak sequence have a global effect on translation efficiency. We found a highly significant and large effect of the nucleotides at position −3 and at position −2 on translation efficiency (Bonferroni adjusted Kruskal–Wallis test *P* = 5.7 × 10^−20^ for position −3; *P* = 1.2 × 10^−17^ at position −2) (Fig. 5A; Supplemental Fig. S5A). Additionally, the 2 nt immediately after the start codon had statistically significant effects on translation efficiency (Bonferroni adjusted *P* < 2.8 × 10^−7^) (Supplemental Fig. S5A). Although previous work using reporter systems anticipated the significance of these features (e.g., Kozak 1987), our analyses highlight the role of sequence composition near the ATG in modulating translation efficiency of endogenous genes at a global scale.

The extent and potential role of natural variation that might alter the Kozak sequence across the genome remains largely unexplored in the human population (Supplemental Table S6; Xu et al. 2010). Among the set of individuals studied here, there were approximately 150 genetic variants altering the Kozak region in at least three individuals. Approximately 65% of Kozak region variants reduced the position weight matrix (PWM) score of the reference sequence (Supplemental Fig. S5B). This effect was even more pronounced for Kozak variants that were observed in a single individual. Of these, 77% reduced the PWM score of the reference sequence, suggesting that selective pressure may be acting to optimize the Kozak sequence.

We next tested the effect of these variants on ribosome occupancy of the main coding region. We utilized the position weight matrix for the Kozak region to score the impact of each variant on the Kozak strength (Fig. 5A). We found nine genes with Kozak variants that modified ribosome occupancy significantly with no significant effect on the RNA levels (10% FDR using Benjamini-Hochberg correction; RNA expression association *P*-value > 0.01; using a conservative linear mixed model, two of these genes had *P* < 0.01) (Fig. 5B,C; Supplemental Fig. S5C), indicating the presence of variants specifically affecting translation efficiency.

Finally, to directly examine the role of genetic variation on translation efficiency, we used reporter assays (Jang et al. 1988) for six genes. These included four genes with Kozak region variants and two genes with uORF variants. We cloned the reference 5′ UTR or the variant 5′ UTR with a single base change at the Kozak region or the uORF upstream of a *Renilla* luciferase and transfected the resulting constructs into human HEK 293 cells. To normalize differences in RNA expression and transfection efficiency, an HCV internal ribosome entry site driven firefly luciferase was cloned after the *Renilla* stop codon, and the ratio of the *Renilla* to firefly luciferase was quantified. Differences in this ratio between the reference and variant 5′-UTR-containing reporters for four genes recapitulated the results from our ribosome profiling data, i.e., sequences that were associated with reduced translational efficiency also gave low luciferase ratios. These results provide an independent validation of our conclusion that natural genetic variation can modify sequences surrounding the start codon leading to personal differences in translation (Fig. 5D–F; Supplemental Fig. S5D).

## Discussion

This study demonstrates that translation efficiency varies among individuals, and nucleotides important for regulating translation efficiency can be identified. In several cases, we uncovered the mechanisms controlling translation efficiency variation in humans. These included uORFs and sequences near the translation initiation sites. Our study revealed that genetic differences between individuals could lead to gene expression differences at the level of translation.

We leveraged replicate measurements to identify genes with significant variability in RNA expression or ribosome occupancy between individuals. We found that genes that exhibit significant variability in both RNA expression and ribosome occupancy were highly enriched for functions directly pertinent to LCLs such as immune response and leukocyte migration (Fig. 3). Hence, extending this analysis framework to additional cell types or tissues will likely uncover more genes with variable expression among individuals.

We also investigated the relationship between differences in protein levels and variability in RNA expression and translation. We found that joint analysis of RNA expression and translation improved our ability to identify the extent of gene expression variation that would be reflected in protein levels, indicating a tight coupling of translation efficiency and protein levels. These analyses were skewed toward genes with higher expression levels due to missing protein level measurements (Supplemental Fig. S3B). Hence, further improvements in proteomics technology will be needed to test the generalizability of our results to lowly expressed proteins. Despite the significant improvements obtained by joint analysis of ribosome occupancy and RNA level measurements, there remains unexplained variability in protein levels. One potential contributor to this discrepancy is variability in protein degradation rates (Vogel and Marcotte 2012). Another important future direction will be to investigate the contribution of RNA sequence features (e.g., Vogel et al. 2010) to the relationship between RNA expression, ribosome occupancy, and protein levels.

Importantly, genes that have individual variability only in RNA expression are less likely to have corresponding differences at the protein level. Among this subset of genes, only 40% had statistically significant covariation between RNA levels and protein levels (5% FDR). An important implication of this result concerns ongoing efforts that aim to identify genetic determinants of RNA expression (Lappalainen et al. 2013; Battle et al. 2014). These studies are in part motivated by the finding that most disease risk factors identified by genome-wide association studies lie in noncoding regions (Edwards et al. 2013). By linking genetic differences to RNA expression, these studies hope to uncover functional connections to disease states. Yet, our analyses suggest that the functional impact of RNA-level differences needs to be carefully considered to establish causal relationships to phenotype.

Recent consortium efforts measured RNA expression in large sets of genotyped samples (approximately 900 in Battle et al. 2014 and approximately 500 samples in Lappalainen et al. 2013) to identify *trans*-acting genetic effects on RNA expression. Interestingly, 85% of the *trans*-effects on RNA expression were mediated by the effects of the associated SNP on a nearby gene (Battle et al. 2014), indicating that changes in regulators of RNA expression lead to differences in RNA levels of distant transcripts. Similarly, genetic variation in translation regulators is likely to have *trans*-effects on ribosome occupancy of many transcripts. For example, levels of global regulators of translation such as MTOR, and translation initiation factors (e.g., EIF4E), have the potential to modulate the translation of a large number of targets (Mamane et al. 2007; Thoreen et al. 2012). In fact, a recent study that compared translation in two different strains of yeast suggested that the relative importance of *trans*-effects on translation is comparable to that for RNA levels (Albert et al. 2014). Future studies in the human population will likely uncover *trans*-acting and additional *cis*-acting genetic variants associated with translation and reveal the contribution of population-level variation to translation variability.

A recent analysis (Battle et al. 2015) of RNA expression, ribosome occupancy, and protein measurements from several human LCLs concluded that there is a scarcity of human genetic variants associated with translation-specific effects. However, we note critical limitations in their ribosome profiling data. As demonstrated in Figure 1 and recently by Miettinen and Björklund (2015), the nuclease digestion conditions used in Battle et al. (2015) lead to severe ribosomal degradation and significantly lower monosome purity in ribosome profiling libraries. Moreover, the Battle et al. (2015) study design lacks replicate experiments, precluding proper assessment of the reproducibility of ribosome profiling measurements. In resequencing experiments, Battle et al. (2015) reported rank correlations lower than 0.9 (Spearman ρ) for the majority of their samples (Supplemental Fig. S2A in Battle et al. 2015). In contrast, we consistently achieved greater than 0.98 rank correlations between biological replicates using independently grown cells and independently prepared ribosome profiling libraries. Here, by leveraging higher quality ribosome profiling data sets with replicates and independent reporter experiments, we identify genetic variants associated with translation efficiency undetected in Battle et al. (2015).

Our study revealed several genetic variants that control translation efficiency variation in humans, including those affecting the Kozak region and upstream open reading frames (uORFs). A particularly interesting question is the molecular mechanisms of these sequence-function relationships. An intriguing feature of genetic variants modifying uORFs on translation was the observation that both gain and loss events could lead to increased translation of the downstream open reading frame (Supplemental Fig. S4G), consistent with previous work that implicated both positive and negative regulation of translation efficiency by uORFs (Brar et al. 2012; Waern and Snyder 2013). Whereas several mechanisms have been implicated in negative regulation of translation efficiency by uORFs, including nonsense mediated decay (Kervestin and Jacobson 2012), less is known about the mechanisms of positive regulation by uORFs. Recent work identified a complex, DENR-MCT1, that catalyzes translation reinitiation downstream from certain uORFs (Schleich et al. 2014), suggesting that DENR-MCT1 or similar factors may act on subsets of uORFs to increase reinitiation frequency of the downstream ORFs leading to higher translation efficiency.

Recent structural analysis of the yeast 48S translation initiation complex permitted an unprecedented view of the molecular environment of the start codon in eukaryotes (Hussain et al. 2014), revealing a potential mechanism by which Kozak region variants affect translation efficiency. Remarkably, this structural analysis revealed that eIF2A directly contacts nucleotides at positions −2 and −3, the same two positions that our global analysis of Kozak variants highlighted as being the most important for translation efficiency (Fig. 5A). Thus, our results provide functional evidence that these residues are of general importance for translational efficiency.

Together, these results demonstrate that genetic alterations in the human population and disease-associated mutations may penetrate to phenotype through changes in translation. In the era of personal genome sequencing, this information is crucial for understanding the role of genetic variants on gene expression, phenotypic traits, and human disease susceptibility.

## Methods

### RNA-seq experiments and ribosome profiling experiments

Human lymphoblastoid cell lines (LCLs) were obtained from Coriell Cell Repository. For replicate ribosome profiling experiments, cells were grown separately to a density of 0.8–1.0 × 10^6^ cells/mL. Approximately 10 million cells were pelleted at 250*g* at 4°C and washed with PBS. The pellets were frozen in liquid nitrogen prior to cell lysis. Seven A260 units of the cleared cell lysates were incubated with 300 units of RNase T1 (Fermentas) and 500 ng of RNase A (Ambion). A 34% (Weight/Volume) sucrose cushion was used to isolate ribosomes. Library preparation and sequencing was done as previously described with some modifications (Supplemental Methods; Ingolia et al. 2012).

For RNA-seq experiments, LCLs were grown to a density of 3 × 10^5^–6 × 10^5^ cells/mL. Total RNA was extracted using TRIzol reagent according to the manufacturer's instructions (Life Technologies), then purified using the Qiagen RNeasy kit (Qiagen) and treated with RNase-free DNase (Qiagen). RNA integrity was checked with a Bioanalyzer (Agilent), and only samples with an RNA integrity number (RIN) of >9.5 were subsequently subjected to either ribosomal depletion or poly-A selection. For ribosomal RNA depletion, 5 μg of purified total RNA was depleted of rRNAs using the Ribo-Zero Magnetic Gold Kit (Human/Mouse/Rat) (Epicentre Biotechnologies). For poly-A selection, 10 μg of purified total RNA were enriched by performing two cycles of selection using the Dynabeads mRNA Purification Kit (Life Technologies). Stranded libraries were prepared following the dUTP protocol (Parkhomchuk et al. 2009). For each cell line, we generated 2 × 101 bp paired-end RNA-seq data using two biological replicates of ribosomal RNA depleted and three biological replicates of poly-A-selected RNA.

### Sequence alignment and processing

To enable comparable analysis of high-throughput sequencing data sets, we used a uniform alignment and preprocessing pipeline. Reads were sequentially aligned using Bowtie 2 v.2.0.5 (Langmead and Salzberg 2012). All reads mapping to human rRNA and tRNA sequences were filtered out. The remaining reads were aligned to APPRIS principal transcripts (release 12) (Rodriguez et al. 2013) from the GENCODE mRNA annotation v.15 (Harrow et al. 2012). For all transcript level analyses, reads that map only to coding regions were used. For details, see Supplemental Methods.

### Ribosome profiling sample identity verification

The cell line identity for all ribosomal profiling libraries were verified by comparing empirically generated genotype calls to the reference genotypes. Specifically, we utilized SAMtools mpileup utility in combination with BCFtools (Li et al. 2009) to generate genotype calls from the ribosomal profiling read alignments. Finally, a custom Perl script was used to compare the number of perfect matches between empirically called genotypes and the reference genotype that was available from the HapMap and the 1000 Genomes Project (The International HapMap 3 Consortium 2010; The 1000 Genomes Project Consortium 2012). For details, see Supplemental Methods.

### Genotype data and processing

Genome sequences were obtained from the 1000 Genomes Project pilot 2 trios and Phase1v3 (The International HapMap 3 Consortium 2010; The 1000 Genomes Project Consortium 2012) for 27 of the 30 individuals. The genome sequences of three cell lines (NA19139, NA19193, and NA19201) were imputed from HapMap release 28 data (The International HapMap Consortium 2007; The International HapMap 3 Consortium 2010) to the 1000 Genomes Phase1v3 reference panel (The 1000 Genomes Project Consortium 2012).

We included all variant calls provided by both release and pilot data sets without additional score or source filtering. We subsetted all single nucleotide polymorphisms (SNPs) that overlap APPRIS transcripts and retained all phasing information from the VCF files. About 8% of the variants in the pilot data set were unphased; and for these variants, we randomly assigned the phase. For details, see Supplemental Methods.

### Sequence data normalization and quality control

After accounting for differences in the mRNA enrichment method, ∼9600 transcripts had a read count per million reads mapped (cpm) (as implemented in the edgeR package) (McCarthy et al. 2012) greater than one in at least 40 RNA-seq libraries and 36 ribosome profiling libraries. We used trimmed mean of *M*-values to account for differences in library size (Robinson and Oshlack 2010) and estimated the mean to variance relationship in the data using the voom method (Law et al. 2014). We explicitly specified the individual identifier to indicate which libraries were replicates from the same individual while applying the voom method. The inverse variance weights obtained from the voom method were used in all analyses where applicable. For details, see Supplemental Methods.

### Calculation of translation efficiency

When combined with RNA expression measurements, ribosome profiling enables the estimation of translation efficiency by capturing a snapshot of the transcriptome-wide ribosome occupancy. We treated ribosome profiling and RNA-seq as two experimental manipulations of the RNA pool of the cell. Translation efficiency was calculated using a linear model, in which the normalized expression values are dependent on the treatment (RNA-seq or ribosome profiling) and the individual identifiers (limma R package) (Smyth 2005). For details, see Supplemental Methods.

### Self-organizing maps for integrative gene expression analysis

We used SOMs to explore the relationship between protein levels and the three expression measurements: RNA levels, ribosome occupancy, and translation efficiency. SOMs rely on a suitable measure of distance between the transcripts for the clustering. To avoid skewing distance calculation due to difference in scale and variance of the expression measurements, expression levels and protein amounts were converted to percentiles using the empirical cumulative distribution function for each level. The kohonen R package (Wehrens and Buydens 2007) was used for training the SOM with custom modifications to the plotting functions following (Xie et al. 2013). We then clustered the codebook vectors of the 140 units in the SOM using affinity propagation clustering (Frey and Dueck 2007) as implemented in the apcluster R package (Bodenhofer et al. 2011). For details, see Supplemental Methods.

### Gene set enrichment analysis

FuncAssociate 2.0 was used for gene set enrichment analyses (Berriz et al. 2009). The background gene list was explicitly defined as the set of all genes that could potentially be included in the query set. We defined significant enrichments as GO terms with an odds ratio >2 and adjusted *P*-value < 0.05. *P*-value adjustment was carried out using a permutation method to account for the overlap between the GO terms. We calculated the Kappa Similarity Score between all pairs of significantly enriched GO terms. We retained edges between all pairwise GO terms whose Kappa similarity score was >0.1. Enriched GO terms were visualized with Cytoscape (Smoot et al. 2011) using the edge-weighted spring embedded layout. For details, see Supplemental Methods.

### Analysis of inter-individual variation in RNA expression and ribosome occupancy

Replicate measurements for RNA-seq and ribosome profiling were used to determine inter-individual variance while controlling for platform specific variance observed between replicates from the same individual. To decompose these two variance components, we used a linear mixed effects model in which we treated the individual as a random effect. As before, we utilized the inverse variance weights obtained from the voom approach and fitted the model using log-likelihood instead of a restricted maximum likelihood approach. We tested the null hypothesis that the variance of the random effect is zero. Rejection of the null hypothesis implied that there was significant inter-individual variance in the expression of the given transcript. We adopted a simulation-based approach using an exact likelihood ratio test implemented in the RLRsim R package (Scheipl et al. 2008). Multiple-hypothesis correction was applied to RNA expression and ribosome occupancy *P*-values separately using Holm's method. For details, see Supplemental Methods.

### Cis-QTL identification

Association between gene expression and the genotype at each variant position located in the exons of the APPRIS transcripts was tested in the set of 21 unrelated Yoruban individuals using PLINK v1.07 (Purcell et al. 2007). For each transcript, replicate gene expression measurements were averaged for this analysis. The expression values were regressed on variant genotypes assuming an additive genetic model where genotype was coded as 0,1, or 2 copies of the alternate allele and restricting the testing to variants with a minor allele frequency >10% in the 21 unrelated Yoruban individuals.

### Genetic determinants of variability in ribosome occupancy

#### Testing the effect of uORF events on ribosome occupancy

We used AUG and CUG as potential start codons, and UAG, UAA, and UGA as potential stop codons. CUG initiation has been reported in few well-documented cases, such as *FGF2*, *VEGF*, *MYC*, and *MHC class I* transcripts (Hann et al. 1988; Vagner et al. 1996; Meiron et al. 2001; Schwab et al. 2003). Additionally, recent studies mapping genome-wide translation initiation sites have suggested that upstream translation initiates frequently from non-AUG codon, most prominently at CUG sites (Ingolia et al. 2011; Leek et al. 2012).

To group individuals by uORF differences on a given transcript, we first determined all possible combinations of uORF gain/loss events. We then tested whether the copy number of the uORF variants affects ribosome occupancy of the main coding region using two approaches. In the first approach, we used linear regression. In the second, more conservative approach, we fitted a linear mixed model assuming the difference in cell lines is an individual-specific random effect, i.e., treating the different cell lines of the same individual as “technical replicates.” For details, see Supplemental Methods.

### The effect of Kozak region sequence on translation efficiency

We defined the Kozak region as the 6 nt preceding the start codon and the 2 nt following the start codon. We extracted the nucleotide sequence of this region from all annotated APPRIS transcripts and built a PWM, which recapitulated the known Kozak sequence (Fig. 5A). We tested whether the nucleotide content of the Kozak sequence affected translation efficiency using the Kruskal–Wallis test. Specifically, we tested whether transcripts split into four categories based on the nucleotide at a given position has the same translation efficiency. We corrected the *P*-value from this test using Bonferroni correction for the eight tests (number of positions) that were performed.

### Association between Kozak region genetic variants and ribosome occupancy

Next, we collected all SNPs that intersect annotated Kozak regions. We scored the variant and the reference Kozak sequence using the PWM matrix obtained above. We coded each variant by the PWM score change and assumed an additive relationship between different positions in the Kozak region and copy number of the allele. We then tested whether the variants in the Kozak regions affect ribosome occupancy of the main coding region using a linear model. For all Kozak variants affecting ribosome occupancy, we conducted the same association test using RNA expression level as the phenotype. As for the uORF analysis, we deemed RNA association to be not significant if the nominal *P*-value was >0.05 or if the regression coefficient had the opposite sign. For details, see Supplemental Methods.

### Luciferase reporter assays

To assay translation efficiency, we used a bicistronic luciferase reporter construct (Jang et al. 1988). This construct has an SV40 promoter that drives the expression of a bicistronic transcript that includes both the firefly and *Renilla* luciferase. Although the *Renilla* luciferase translation is cap-dependent, firefly luciferase has a Hepatitis C virus (HCV) internal ribosome entry site (IRES) that enables cap-independent translation.

Gene segments were synthesized and cloned right in front of the start codon (ATG) of the *Renilla* luciferase using the CloneEZ system (GenScript). The bicistronic constructs were transfected into HEK293 cells. Cap-dependent translation was calculated by taking the ratio of *Renilla* (cap) to firefly (HCV IRES) luciferase activity and derived from five replicate experiments. The HCV-IRES-dependent translation of firefly luciferase accounted for differences in RNA expression and transfection efficiency. Outlier detection was carried out as described (Jacobs and Dinman 2004). The difference between *Renilla* to firefly luciferase ratios was assessed using a Welch two-sample two-sided *t*-test. For details, see Supplemental Methods.

## Data access

The sequencing data from this study have been submitted to the NCBI Gene Expression Omnibus (GEO; http://www.ncbi.nlm.nih.gov/geo/) under accession number GSE65912.

## Supplementary Material

Supplemental Material

## References

[CENIKGR193342C1] The 1000 Genomes Project Consortium. 2012 An integrated map of genetic variation from 1,092 human genomes. Nature 491: 56–65. 23128226 10.1038/nature11632 PMC3498066

[CENIKGR193342C2] Albert FW, Muzzey D, Weissman JS, Kruglyak L. 2014 Genetic influences on translation in yeast. PLoS Genet 10: e1004692. 25340754 10.1371/journal.pgen.1004692 PMC4207643

[CENIKGR193342C3] Artieri CG, Fraser HB. 2014 Evolution at two levels of gene expression in yeast. Genome Res 24: 411–421. 24318729 10.1101/gr.165522.113 PMC3941106

[CENIKGR193342C4] Barbosa C, Peixeiro I, Romão L. 2013 Gene expression regulation by upstream open reading frames and human disease. PLoS Genet 9: e1003529. 23950723 10.1371/journal.pgen.1003529 PMC3738444

[CENIKGR193342C5] Battle A, Mostafavi S, Zhu X, Potash JB, Weissman MM, McCormick C, Haudenschild CD, Beckman KB, Shi J, Mei R, 2014 Characterizing the genetic basis of transcriptome diversity through RNA-sequencing of 922 individuals. Genome Res 24: 14–24. 24092820 10.1101/gr.155192.113 PMC3875855

[CENIKGR193342C6] Battle A, Khan Z, Wang SH, Mitrano A, Ford MJ, Pritchard JK, Gilad Y. 2015 Impact of regulatory variation from RNA to protein. Science 347: 664–667. 25657249 10.1126/science.1260793 PMC4507520

[CENIKGR193342C12] Benjamini Y, Hochberg Y. 1995 Controlling the false discovery rate: a practical and powerful approach to multiple testing. J R Statist Soc B 57: 289–300.

[CENIKGR193342C7] Berriz GF, King OD, Bryant B, Sander C, Roth FP. 2003 Characterizing gene sets with FuncAssociate. Bioinformatics 19: 2502–2504. 14668247 10.1093/bioinformatics/btg363

[CENIKGR193342C8] Berriz GF, Beaver JE, Cenik C, Tasan M, Roth FP. 2009 Next generation software for functional trend analysis. Bioinformatics 25: 3043–3044. 19717575 10.1093/bioinformatics/btp498 PMC2800365

[CENIKGR193342C9] Bodenhofer U, Kothmeier A, Hochreiter S. 2011 APCluster: an R package for affinity propagation clustering. Bioinformatics 27: 2463–2464. 21737437 10.1093/bioinformatics/btr406

[CENIKGR193342C10] Brar GA, Yassour M, Friedman N, Regev A, Ingolia NT, Weissman JS. 2012 High-resolution view of the yeast meiotic program revealed by ribosome profiling. Science 335: 552–557. 22194413 10.1126/science.1215110 PMC3414261

[CENIKGR193342C11] Calvo SE, Pagliarini DJ, Mootha VK. 2009 Upstream open reading frames cause widespread reduction of protein expression and are polymorphic among humans. Proc Natl Acad Sci 106: 7507–7512. 19372376 10.1073/pnas.0810916106 PMC2669787

[CENIKGR193342C13] Crooks GE, Hon G, Chandonia JM, Brenner SE. 2004 WebLogo: a sequence logo generator. Genome Res 14: 1188–1190. 15173120 10.1101/gr.849004 PMC419797

[CENIKGR193342C15] Darnell JC. 2011 Defects in translational regulation contributing to human cognitive and behavioral disease. Curr Opin Genet Dev 21: 465–473. 21764293 10.1016/j.gde.2011.05.002 PMC3166213

[CENIKGR193342C18] Dunn JG, Foo CK, Belletier NG, Gavis ER, Weissman JS. 2013 Ribosome profiling reveals pervasive and regulated stop codon readthrough in *Drosophila melanogaster*. Elife 2: e01179. 24302569 10.7554/eLife.01179 PMC3840789

[CENIKGR193342C19] Edwards SL, Beesley J, French JD, Dunning AM. 2013 Beyond GWASs: illuminating the dark road from association to function. Am J Hum Genet 93: 779–797. 24210251 10.1016/j.ajhg.2013.10.012 PMC3824120

[CENIKGR193342C20] Frey BJ, Dueck D. 2007 Clustering by passing messages between data points. Science 315: 972–976. 17218491 10.1126/science.1136800

[CENIKGR193342C22] Hann SR, King MW, Bentley DL, Anderson CW, Eisenman RN. 1988 A non-AUG translational initiation in c-*myc* exon 1 generates an N-terminally distinct protein whose synthesis is disrupted in Burkitt's lymphomas. Cell 52: 185–195. 3277717 10.1016/0092-8674(88)90507-7

[CENIKGR193342C23] Harrow J, Frankish A, Gonzalez JM, Tapanari E, Diekhans M, Kokocinski F, Aken BL, Barrell D, Zadissa A, Searle S, 2012 GENCODE: the reference human genome annotation for The ENCODE Project. Genome Res 22: 1760–1774. 22955987 10.1101/gr.135350.111 PMC3431492

[CENIKGR193342C24] Holm S. 1979 A simple sequentially rejective multiple test procedure. Scand J Statist 6: 65–70.

[CENIKGR193342C25] Horn S, Figl A, Rachakonda PS, Fischer C, Sucker A, Gast A, Kadel S, Moll I, Nagore E, Hemminki K, 2013 *TERT* promoter mutations in familial and sporadic melanoma. Science 339: 959–961. 23348503 10.1126/science.1230062

[CENIKGR193342C28] Huang FW, Hodis E, Xu MJ, Kryukov GV, Chin L, Garraway LA. 2013 Highly recurrent *TERT* promoter mutations in human melanoma. Science 339: 957–959. 23348506 10.1126/science.1229259 PMC4423787

[CENIKGR193342C29] Hussain T, Llácer JL, Fernández IS, Munoz A, Martin-Marcos P, Savva CG, Lorsch JR, Hinnebusch AG, Ramakrishnan V. 2014 Structural changes enable start codon recognition by the eukaryotic translation initiation complex. Cell 159: 597–607. 25417110 10.1016/j.cell.2014.10.001 PMC4217140

[CENIKGR193342C30] Ingolia NT, Ghaemmaghami S, Newman JRS, Weissman JS. 2009 Genome-wide analysis in vivo of translation with nucleotide resolution using ribosome profiling. Science 324: 218–223. 19213877 10.1126/science.1168978 PMC2746483

[CENIKGR193342C31] Ingolia NT, Lareau LF, Weissman JS. 2011 Ribosome profiling of mouse embryonic stem cells reveals the complexity and dynamics of mammalian proteomes. Cell 147: 789–802. 22056041 10.1016/j.cell.2011.10.002 PMC3225288

[CENIKGR193342C32] Ingolia NT, Brar GA, Rouskin S, McGeachy AM, Weissman JS. 2012 The ribosome profiling strategy for monitoring translation *in vivo* by deep sequencing of ribosome-protected mRNA fragments. Nat Protoc 7: 1534–1550. 22836135 10.1038/nprot.2012.086 PMC3535016

[CENIKGR193342C33] The International HapMap 3 Consortium. 2010 Integrating common and rare genetic variation in diverse human populations. Nature 467: 52–58. 20811451 10.1038/nature09298 PMC3173859

[CENIKGR193342C34] The International HapMap Consortium. 2007 A second generation human haplotype map of over 3.1 million SNPs. Nature 449: 851–861. 17943122 10.1038/nature06258 PMC2689609

[CENIKGR193342C35] Jacobs JL, Dinman JD. 2004 Systematic analysis of bicistronic reporter assay data. Nucleic Acids Res 32: e160. 15561995 10.1093/nar/gnh157 PMC534638

[CENIKGR193342C36] Jang SK, Kräusslich HG, Nicklin MJ, Duke GM, Palmenberg AC, Wimmer E. 1988 A segment of the 5′ nontranslated region of encephalomyocarditis virus RNA directs internal entry of ribosomes during in vitro translation. J Virol 62: 2636–2643. 2839690 10.1128/jvi.62.8.2636-2643.1988 PMC253694

[CENIKGR193342C37] Kasowski M, Grubert F, Heffelfinger C, Hariharan M, Asabere A, Waszak SM, Habegger L, Rozowsky J, Shi M, Urban AE, 2010 Variation in transcription factor binding among humans. Science 328: 232–235. 20299548 10.1126/science.1183621 PMC2938768

[CENIKGR193342C38] Kasowski M, Kyriazopoulou-Panagiotopoulou S, Grubert F, Zaugg JB, Kundaje A, Liu Y, Boyle AP, Zhang QC, Zakharia F, Spacek DV, 2013 Extensive variation in chromatin states across humans. Science 342: 750–752. 24136358 10.1126/science.1242510 PMC4075767

[CENIKGR193342C40] Kervestin S, Jacobson A. 2012 NMD: a multifaceted response to premature translational termination. Nat Rev Mol Cell Biol 13: 700–712. 23072888 10.1038/nrm3454 PMC3970730

[CENIKGR193342C41] Khan Z, Ford MJ, Cusanovich DA, Mitrano A, Pritchard JK, Gilad Y. 2013 Primate transcript and protein expression levels evolve under compensatory selection pressures. Science 342: 1100–1104. 24136357 10.1126/science.1242379 PMC3994702

[CENIKGR193342C42] Kohonen T. 1990 The self-organizing map. Proc IEEE 78: 1464–1480.

[CENIKGR193342C44] Kozak M. 1987 At least six nucleotides preceding the AUG initiator codon enhance translation in mammalian cells. J Mol Biol 196: 947–950. 3681984 10.1016/0022-2836(87)90418-9

[CENIKGR193342C45] Langmead B, Salzberg SL. 2012 Fast gapped-read alignment with Bowtie 2. Nat Methods 9: 357–359. 22388286 10.1038/nmeth.1923 PMC3322381

[CENIKGR193342C46] Lappalainen T, Sammeth M, Friedländer MR, ’t Hoen PAC, Monlong J, Rivas MA, Gonzàlez-Porta M, Kurbatova N, Griebel T, Ferreira PG, 2013 Transcriptome and genome sequencing uncovers functional variation in humans. Nature 501: 506–511. 24037378 10.1038/nature12531 PMC3918453

[CENIKGR193342C48] Law CW, Chen Y, Shi W, Smyth GK. 2014 voom: precision weights unlock linear model analysis tools for RNA-seq read counts. Genome Biol 15: R29. 24485249 10.1186/gb-2014-15-2-r29 PMC4053721

[CENIKGR193342C49] Leek JT, Johnson WE, Parker HS, Jaffe AE, Storey JD. 2012 The sva package for removing batch effects and other unwanted variation in high-throughput experiments. Bioinformatics 28: 882–883. 22257669 10.1093/bioinformatics/bts034 PMC3307112

[CENIKGR193342C50] Li H, Handsaker B, Wysoker A, Fennell T, Ruan J, Homer N, Marth G, Abecasis G, Durbin R; 1000 Genome Project Data Processing Subgroup. 2009 The Sequence Alignment/Map format and SAMtools. Bioinformatics 25: 2078–2079. 19505943 10.1093/bioinformatics/btp352 PMC2723002

[CENIKGR193342C51] Ly T, Ahmad Y, Shlien A, Soroka D, Mills A, Emanuele MJ, Stratton MR, Lamond AI. 2014 A proteomic chronology of gene expression through the cell cycle in human myeloid leukemia cells. Elife 3: e01630. 24596151 10.7554/eLife.01630 PMC3936288

[CENIKGR193342C52] Mamane Y, Petroulakis E, Martineau Y, Sato TA, Larsson O, Rajasekhar VK, Sonenberg N. 2007 Epigenetic activation of a subset of mRNAs by eIF4E explains its effects on cell proliferation. PLoS One 2: e242. 17311107 10.1371/journal.pone.0000242 PMC1797416

[CENIKGR193342C53] Marguerat S, Schmidt A, Codlin S, Chen W, Aebersold R, Bähler J. 2012 Quantitative analysis of fission yeast transcriptomes and proteomes in proliferating and quiescent cells. Cell 151: 671–683. 23101633 10.1016/j.cell.2012.09.019 PMC3482660

[CENIKGR193342C55] McCarthy DJ, Chen Y, Smyth GK. 2012 Differential expression analysis of multifactor RNA-Seq experiments with respect to biological variation. Nucleic Acids Res 40: 4288–4297. 22287627 10.1093/nar/gks042 PMC3378882

[CENIKGR193342C56] McDaniell R, Lee BK, Song L, Liu Z, Boyle AP, Erdos MR, Scott LJ, Morken MA, Kucera KS, Battenhouse A, 2010 Heritable individual-specific and allele-specific chromatin signatures in humans. Science 328: 235–239. 20299549 10.1126/science.1184655 PMC2929018

[CENIKGR193342C57] McManus CJ, May GE, Spealman P, Shteyman A. 2014 Ribosome profiling reveals post-transcriptional buffering of divergent gene expression in yeast. Genome Res 24: 422–430. 24318730 10.1101/gr.164996.113 PMC3941107

[CENIKGR193342C58] Meiron M, Anunu R, Scheinman EJ, Hashmueli S, Levi BZ. 2001 New isoforms of VEGF are translated from alternative initiation CUG codons located in its 5′UTR. Biochem Biophys Res Commun 282: 1053–1060. 11352659 10.1006/bbrc.2001.4684

[CENIKGR193342C59] Miettinen TP, Björklund M. 2015 Modified ribosome profiling reveals high abundance of ribosome protected mRNA fragments derived from 3′ untranslated regions. Nucleic Acids Res 43: 1019–1034. 25550424 10.1093/nar/gku1310 PMC4333376

[CENIKGR193342C60] Montgomery SB, Sammeth M, Gutierrez-Arcelus M, Lach RP, Ingle C, Nisbett J, Guigo R, Dermitzakis ET. 2010 Transcriptome genetics using second generation sequencing in a Caucasian population. Nature 464: 773–777. 20220756 10.1038/nature08903 PMC3836232

[CENIKGR193342C61] Muzzey D, Sherlock G, Weissman JS. 2014 Extensive and coordinated control of allele-specific expression by both transcription and translation in *Candida albicans*. Genome Res 24: 963–973. 24732588 10.1101/gr.166322.113 PMC4032860

[CENIKGR193342C63] Pai AA, Cain CE, Mizrahi-Man O, De Leon S, Lewellen N, Veyrieras JB, Degner JF, Gaffney DJ, Pickrell JK, Stephens M, 2012 The contribution of RNA decay quantitative trait loci to inter-individual variation in steady-state gene expression levels. PLoS Genet 8: e1003000. 23071454 10.1371/journal.pgen.1003000 PMC3469421

[CENIKGR193342C64] Parkhomchuk D, Borodina T, Amstislavskiy V, Banaru M, Hallen L, Krobitsch S, Lehrach H, Soldatov A. 2009 Transcriptome analysis by strand-specific sequencing of complementary DNA. Nucleic Acids Res 37: e123. 19620212 10.1093/nar/gkp596 PMC2764448

[CENIKGR193342C65] Pickrell JK, Marioni JC, Pai AA, Degner JF, Engelhardt BE, Nkadori E, Veyrieras JB, Stephens M, Gilad Y, Pritchard JK. 2010 Understanding mechanisms underlying human gene expression variation with RNA sequencing. Nature 464: 768–772. 20220758 10.1038/nature08872 PMC3089435

[CENIKGR193342C66] Purcell S, Neale B, Todd-Brown K, Thomas L, Ferreira MA, Bender D, Maller J, Sklar P, de Bakker PI, Daly MJ, 2007 PLINK: a tool set for whole-genome association and population-based linkage analyses. Am J Hum Genet 81: 559–575. 17701901 10.1086/519795 PMC1950838

[CENIKGR193342C68] Ricci EP, Kucukural A, Cenik C, Mercier BC, Singh G, Heyer EE, Ashar-Patel A, Peng L, Moore MJ. 2014 Staufen1 senses overall transcript secondary structure to regulate translation. Nat Struct Mol Biol 21: 26–35. 24336223 10.1038/nsmb.2739 PMC4605437

[CENIKGR193342C69] Robinson MD, Oshlack A. 2010 A scaling normalization method for differential expression analysis of RNA-seq data. Genome Biol 11: R25. 20196867 10.1186/gb-2010-11-3-r25 PMC2864565

[CENIKGR193342C70] Rodriguez JM, Maietta P, Ezkurdia I, Pietrelli A, Wesselink JJ, Lopez G, Valencia A, Tress ML. 2013 APPRIS: annotation of principal and alternative splice isoforms. Nucleic Acids Res 41: D110–D117. 23161672 10.1093/nar/gks1058 PMC3531113

[CENIKGR193342C71] Scheipl F, Greven S, Küchenhoff H. 2008 Size and power of tests for a zero random effect variance or polynomial regression in additive and linear mixed models. Comput Stat Data Anal 52: 3283–3299.

[CENIKGR193342C72] Schleich S, Strassburger K, Janiesch PC, Koledachkina T, Miller KK, Haneke K, Cheng YS, Küchler K, Stoecklin G, Duncan KE, 2014 DENR–MCT-1 promotes translation re-initiation downstream of uORFs to control tissue growth. Nature 512: 208–212. 25043021 10.1038/nature13401 PMC4134322

[CENIKGR193342C67] Schwab SR, Li KC, Kang C, Shastri N. 2003 Constitutive display of cryptic translation products by MHC class I molecules. Science 301: 1367–1371. 12958358 10.1126/science.1085650

[CENIKGR193342C73] Schwanhäusser B, Busse D, Li N, Dittmar G, Schuchhardt J, Wolf J, Chen W, Selbach M. 2011 Global quantification of mammalian gene expression control. Nature 473: 337–342. 21593866 10.1038/nature10098

[CENIKGR193342C74] Smoot ME, Ono K, Ruscheinski J, Wang PL, Ideker T. 2011 Cytoscape 2.8: new features for data integration and network visualization. Bioinformatics 27: 431–432. 21149340 10.1093/bioinformatics/btq675 PMC3031041

[CENIKGR193342C75] Smyth GK. 2005 Limma: linear models for microarray data. In Bioinformatics and computational biology solutions using R and bioconductor (ed. Gentleman R, ), pp. 397–420. Springer, New York.

[CENIKGR193342C77] Stranger BE, Forrest MS, Dunning M, Ingle CE, Beazley C, Thorne N, Redon R, Bird CP, de Grassi A, Lee C, 2007 Relative impact of nucleotide and copy number variation on gene expression phenotypes. Science 315: 848–853. 17289997 10.1126/science.1136678 PMC2665772

[CENIKGR193342C78] ’t Hoen PA, Friedländer MR, Almlöf J, Sammeth M, Pulyakhina I, Anvar SY, Laros JF, Buermans HP, Karlberg O, Brännvall M, 2013 Reproducibility of high-throughput mRNA and small RNA sequencing across laboratories. Nat Biotechnol 31: 1015–1022. 24037425 10.1038/nbt.2702

[CENIKGR193342C79] Thoreen CC, Chantranupong L, Keys HR, Wang T, Gray NS, Sabatini DM. 2012 A unifying model for mTORC1-mediated regulation of mRNA translation. Nature 485: 109–113. 22552098 10.1038/nature11083 PMC3347774

[CENIKGR193342C80] Vagner S, Touriol C, Galy B, Audigier S, Gensac MC, Amalric F, Bayard F, Prats H, Prats AC. 1996 Translation of CUG- but not AUG-initiated forms of human fibroblast growth factor 2 is activated in transformed and stressed cells. J Cell Biol 135: 1391–1402. 8947560 10.1083/jcb.135.5.1391 PMC2121090

[CENIKGR193342C81] Vogel C, Marcotte EM. 2012 Insights into the regulation of protein abundance from proteomic and transcriptomic analyses. Nat Rev Genet 13: 227–232. 22411467 10.1038/nrg3185 PMC3654667

[CENIKGR193342C82] Vogel C, Abreu Rde S, Ko D, Le SY, Shapiro BA, Burns SC, Sandhu D, Boutz DR, Marcotte EM, Penalva LO. 2010 Sequence signatures and mRNA concentration can explain two-thirds of protein abundance variation in a human cell line. Mol Syst Biol 6: 400. 20739923 10.1038/msb.2010.59 PMC2947365

[CENIKGR193342C83] Waern K, Snyder M. 2013 Extensive transcript diversity and novel upstream open reading frame regulation in yeast. G3 3: 343–352. 23390610 10.1534/g3.112.003640 PMC3564994

[CENIKGR193342C84] Wehrens R, Buydens L. 2007 Self- and super-organizing maps in R: the kohonen package. J Stat Softw 21: 1–19.

[CENIKGR193342C85] Wen Y, Liu Y, Xu Y, Zhao Y, Hua R, Wang K, Sun M, Li Y, Yang S, Zhang XJ, 2009 Loss-of-function mutations of an inhibitory upstream ORF in the human hairless transcript cause Marie Unna hereditary hypotrichosis. Nat Genet 41: 228–233. 19122663 10.1038/ng.276

[CENIKGR193342C86] Westra HJ, Peters MJ, Esko T, Yaghootkar H, Schurmann C, Kettunen J, Christiansen MW, Fairfax BP, Schramm K, Powell JE, 2013 Systematic identification of *trans* eQTLs as putative drivers of known disease associations. Nat Genet 45: 1238–1243. 24013639 10.1038/ng.2756 PMC3991562

[CENIKGR193342C87] Wethmar K, Barbosa-Silva A, Andrade-Navarro MA, Leutz A. 2014 uORFdb—a comprehensive literature database on eukaryotic uORF biology. Nucleic Acids Res 42: D60–D67. 24163100 10.1093/nar/gkt952 PMC3964959

[CENIKGR193342C88] Wu L, Candille SI, Choi Y, Xie D, Jiang L, Li-Pook-Than J, Tang H, Snyder M. 2013 Variation and genetic control of protein abundance in humans. Nature 499: 79–82. 23676674 10.1038/nature12223 PMC3789121

[CENIKGR193342C89] Xie D, Boyle AP, Wu L, Zhai J, Kawli T, Snyder M. 2013 Dynamic *trans*-acting factor colocalization in human cells. Cell 155: 713–724. 24243024 10.1016/j.cell.2013.09.043 PMC4079469

[CENIKGR193342C90] Xu H, Wang P, You J, Zheng Y, Fu Y, Tang Q, Zhou L, Wei Z, Lin B, Shu Y, 2010 Screening of Kozak-motif-located SNPs and analysis of their association with human diseases. Biochem Biophys Res Commun 392: 89–94. 20059968 10.1016/j.bbrc.2010.01.002

