# Supplementary material for: Integrative analysis of RNA, translation, and protein levels reveals distinct regulatory variation across humans
Source: Genome Res. 2015 Nov;25(11):1610–21. doi: 10.1101/gr.193342.115 (PMC4617958; doi:10.1101/gr.193342.115)
Supplement: Supplemental Material [file supp_25_11_1610__index.html]

Supplemental Material 

# Integrative analysis of RNA, translation and protein levels reveals distinct regulatory variation across humans

## Supplemental Material

**Files in this Data Supplement:**

- FigureS1.pdf
- FigureS2.pdf
- FigureS3.pdf
- FigureS4.pdf
- FigureS5.pdf
- Supplemental Material.docx
- Table S1.xlsx
- Table S2.xlsx
- Table S3.xlsx
- Table S4.xlsx
- Table S5.xlsx
- Table S6.xlsx
- Supplemental Figure Table and Legends.docx
