# Supplementary figures and images for: Integrative analysis of RNA, translation, and protein levels reveals distinct regulatory variation across humans
Source: Genome Res. 2015 Nov;25(11):1610–21. doi: 10.1101/gr.193342.115 (PMC4617958; doi:10.1101/gr.193342.115)

Figure S1

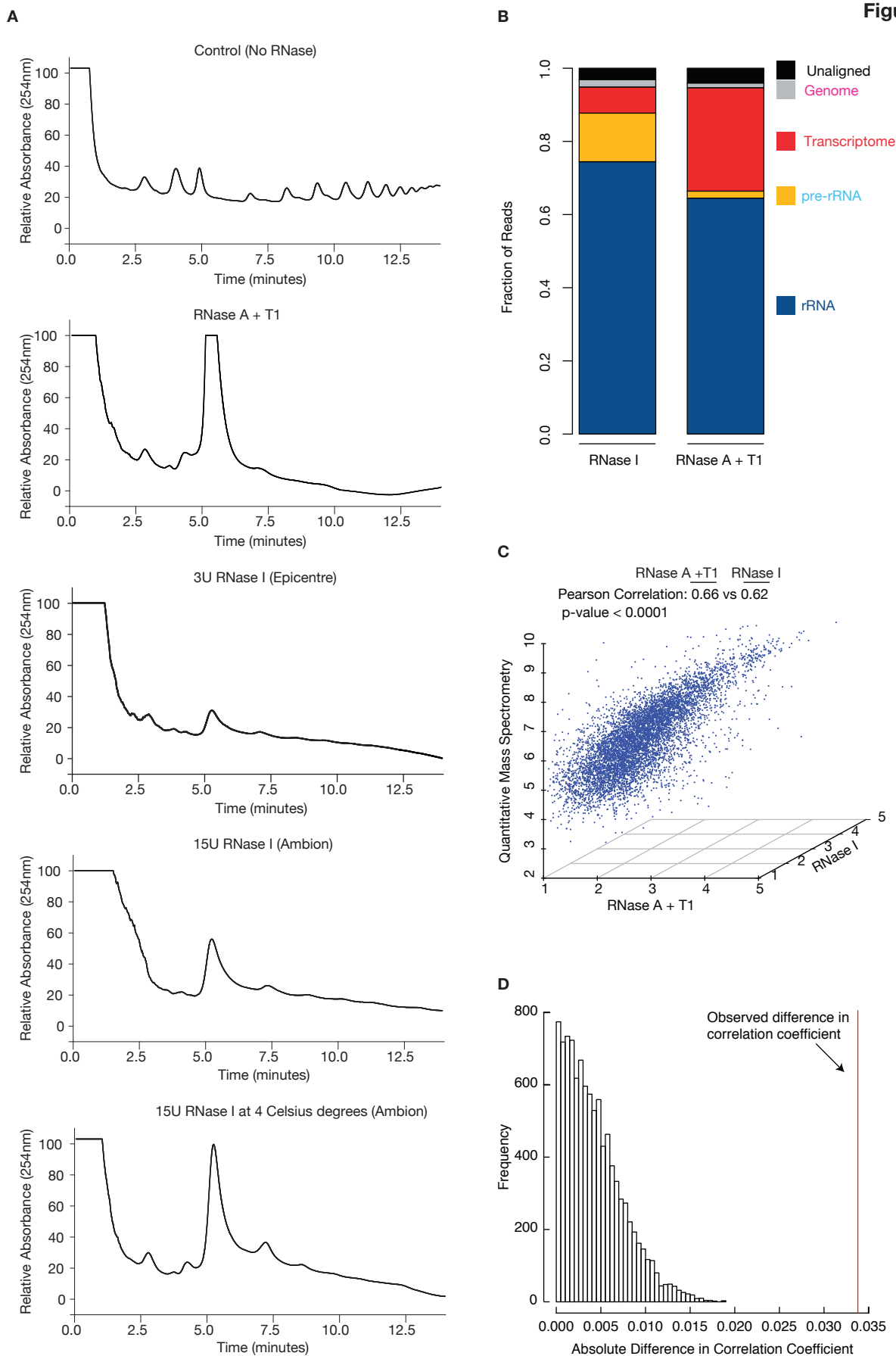

Supplement: Supplemental Material [file supp_gr.193342.115_FigureS1.pdf]

Figure S2

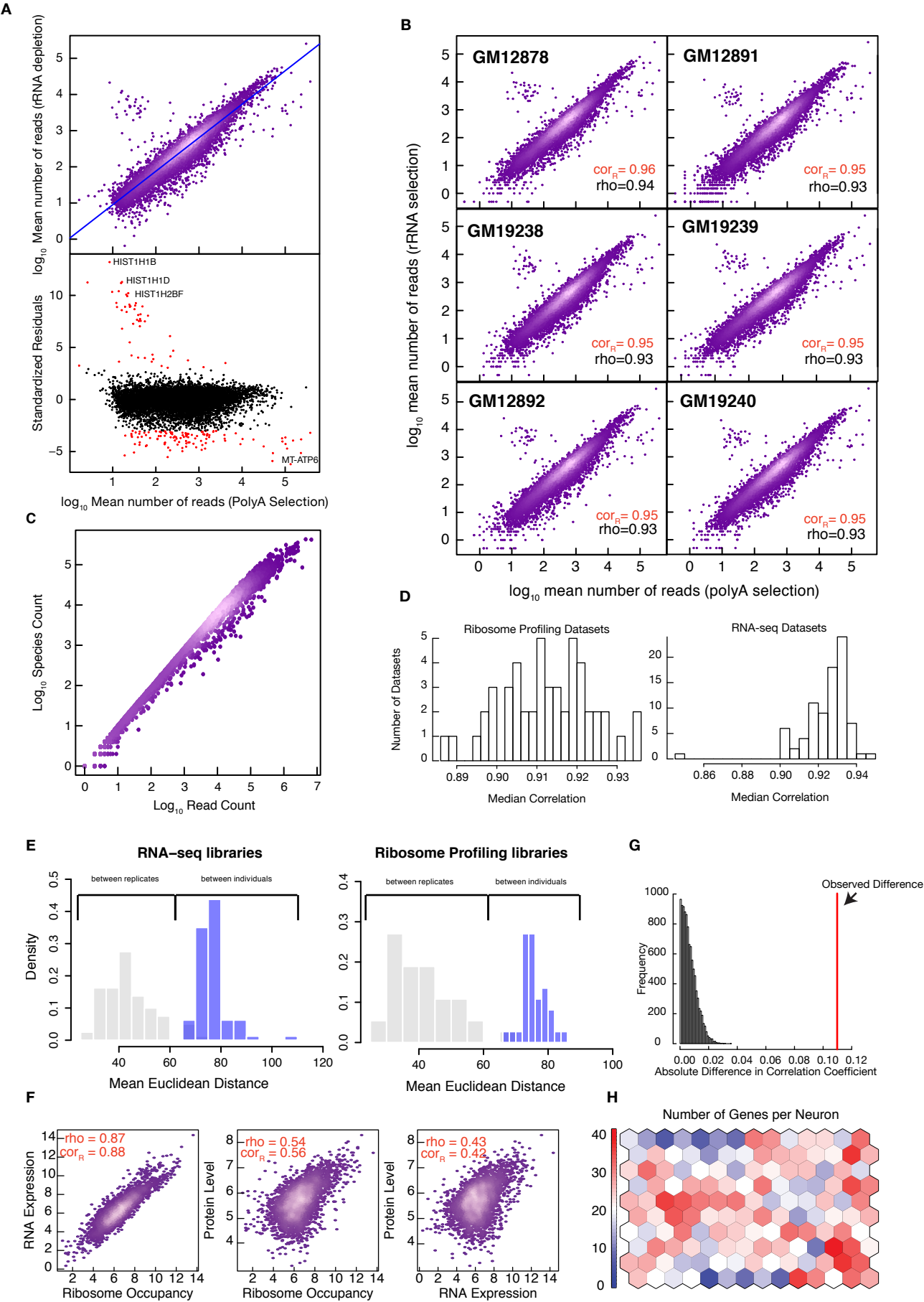

Supplement: Supplemental Material [file supp_gr.193342.115_FigureS2.pdf]

Figure S3

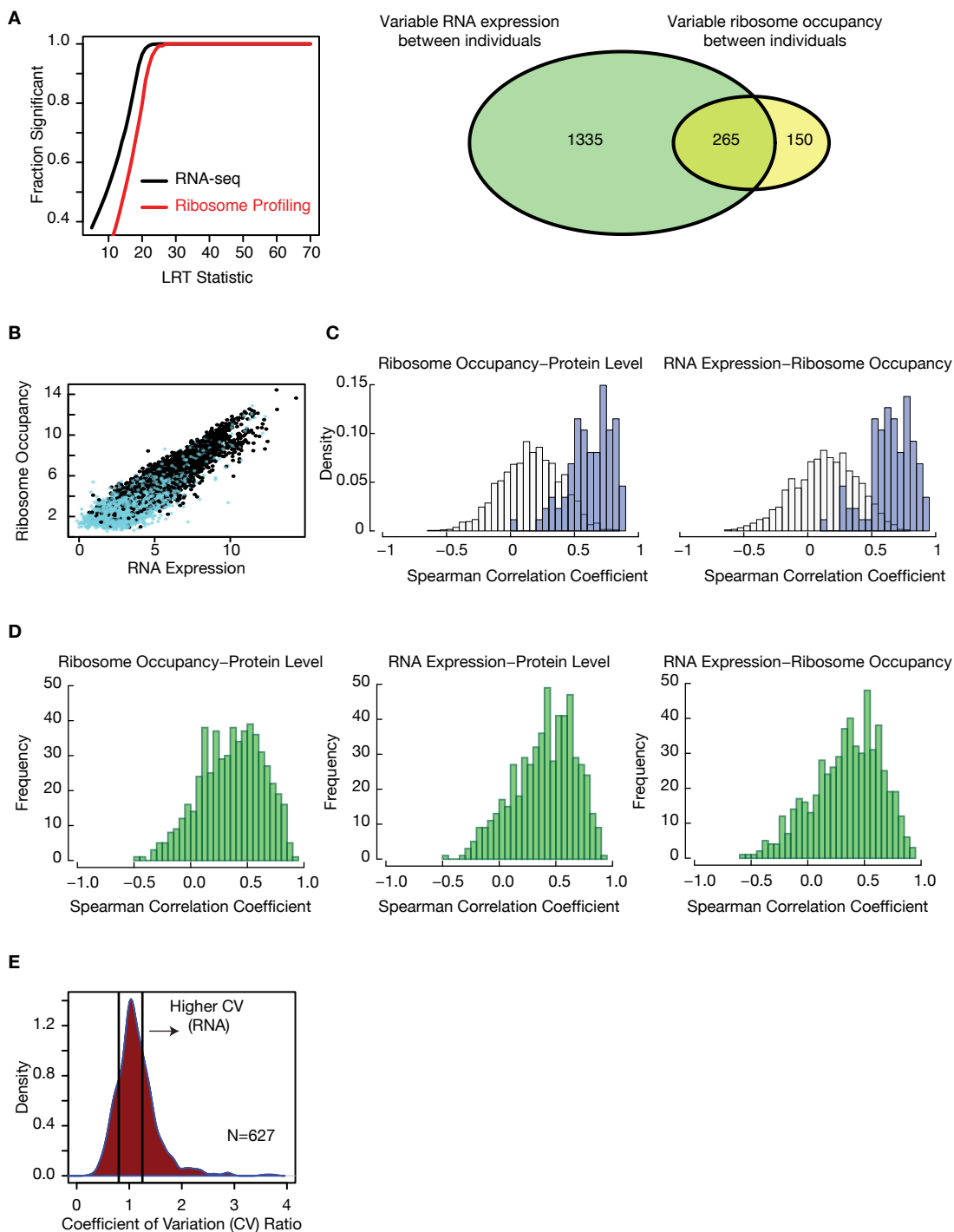

Supplement: Supplemental Material [file supp_gr.193342.115_FigureS3.pdf]

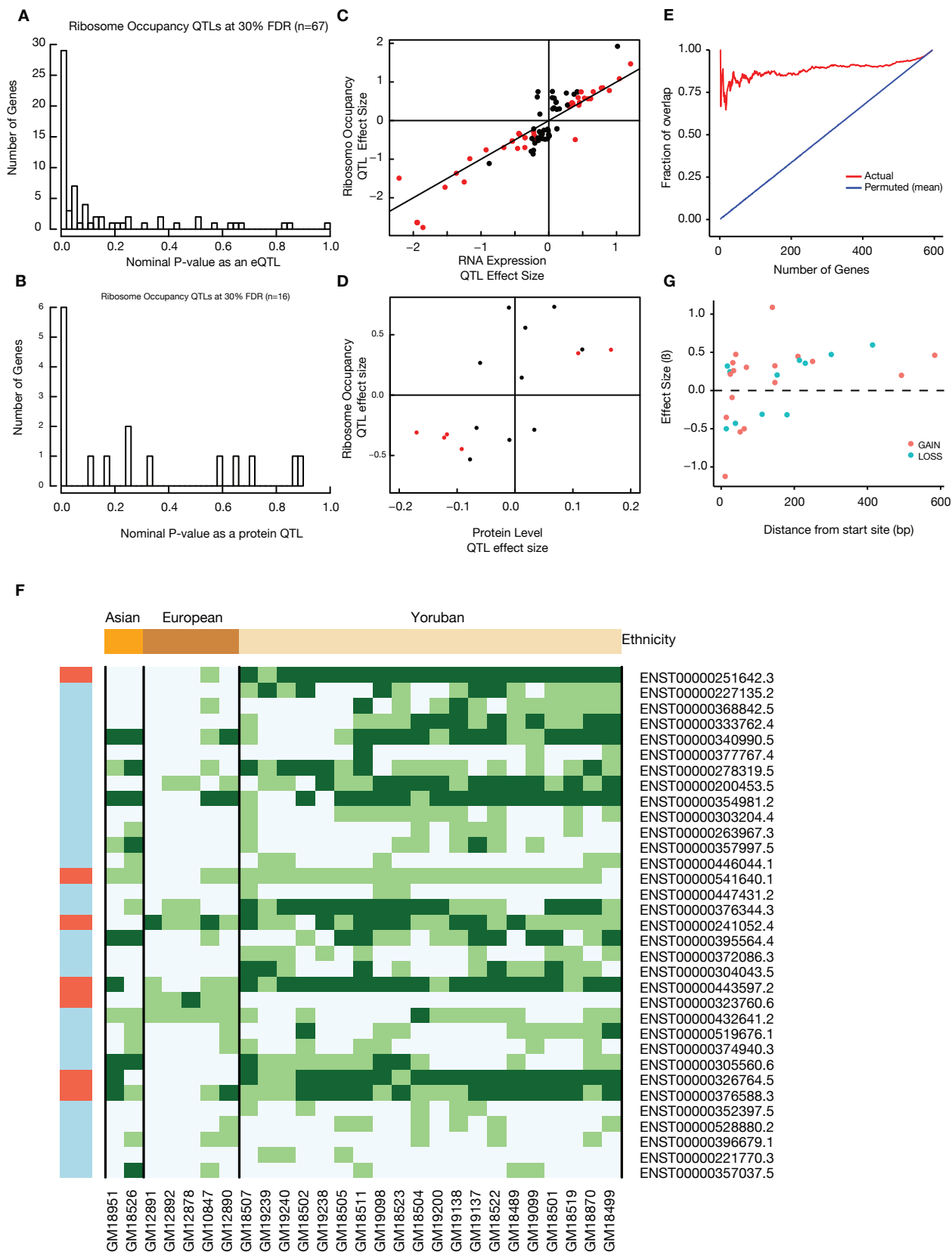

Supplement: Supplemental Material [file supp_gr.193342.115_FigureS4.pdf]

Figure S5

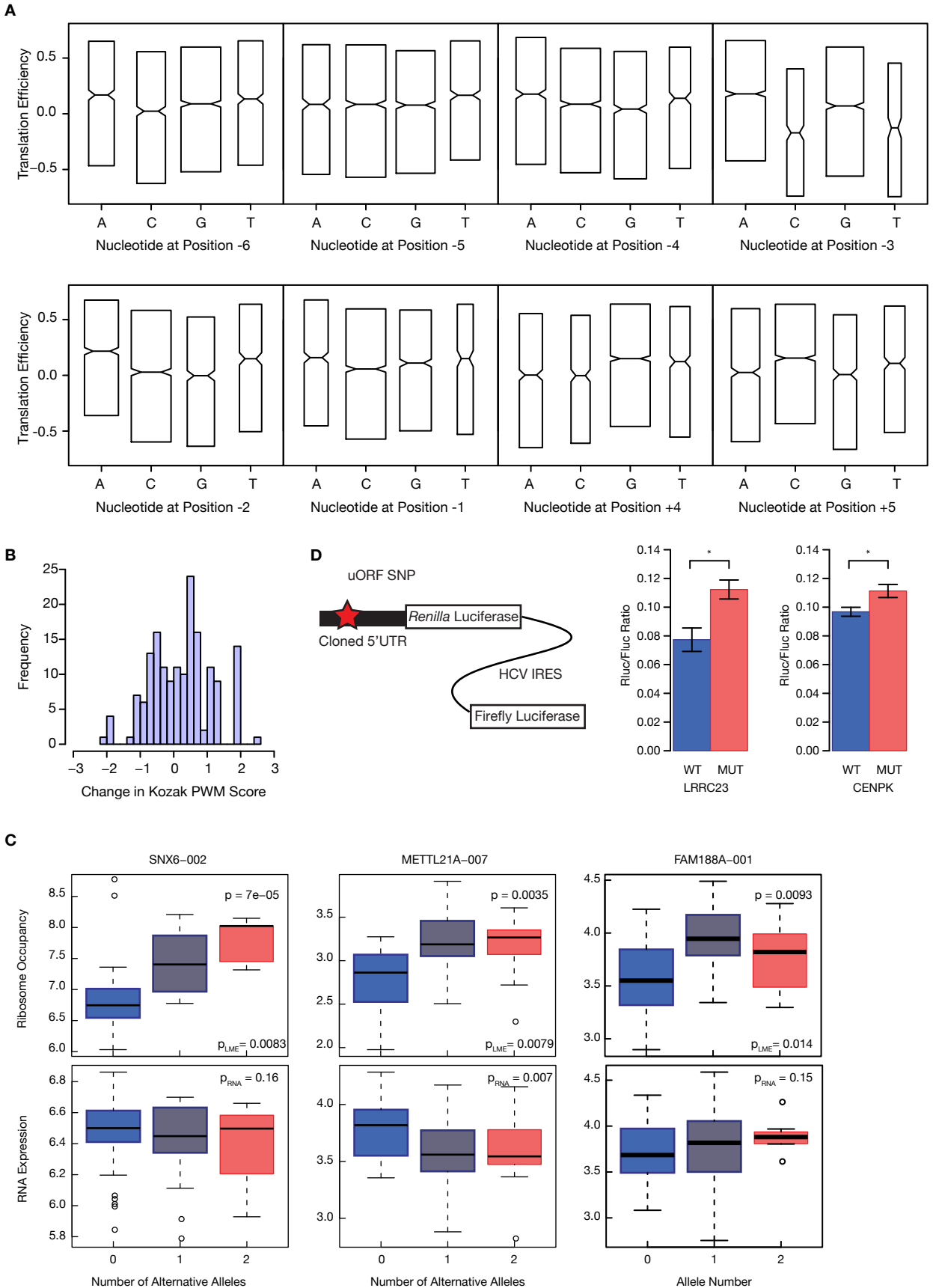

Supplement: Supplemental Material [file supp_gr.193342.115_FigureS5.pdf]
