# Supplementary material for: Integrative analysis of RNA, translation, and protein levels reveals distinct regulatory variation across humans
Source: Genome Res. 2015 Nov;25(11):1610–21. doi: 10.1101/gr.193342.115 (PMC4617958; doi:10.1101/gr.193342.115)
Supplement: Supplemental Material [file supp_gr.193342.115_Supplemental_Material.docx]

**Supplemental Methods:**

**Cell culture and lysis**

Human lymphoblastoid cell lines (LCLs) were obtained from Coriell Cell Repository and grown in 15% fetal bovine serum and 1% Pen-Strep. For replicate experiments, cells were grown separately to a density of 0.8-1.0 x 10^6^ cells/mL. Approximately 10 million cells were pelleted at 250g at 4C and washed with PBS. The pellets were frozen in liquid nitrogen prior to cell lysis. Cells were lysed in 150μl of lysis buffer (20 mM Tris-HCl pH 7.5; 150 mM NaCl; 5mM MgCl2; 1mM DTT; 100 μg.ml^-1^ Cycloheximide; 1% Triton X-100; 25U.ml^-1^ Turbo DNase I). Lysate was homogenized using a P1000 pipettor and gently pipetting up and down. Lysate was then incubated at 4ºC for 10 minutes before centrifugation at 1300g for 10 minutes at 4ºC. The supernatant was recovered and absorbance at 260nm measured.

HEK293 cells were treated similarly with the following differences. Cells were seeded at a density of 5 million cells per 150mm plate. After 16 hours of culture, cycloheximide was added to 100 μg.ml^-1^ for 10 minutes. Cells were then washed two times in ice-cold PBS with cycloheximide (100 μg.ml^-1^) and scraped in 1ml of PBS with cycloheximide (100 μg.ml^-1^). Cells were then pelleted at 500g for 5 minutes at 4ºC and lysed in 1ml of lysis buffer (10mM Tris-HCl pH. 7.5; 5mM MgCl2; 100mM KCl; 1% Triton X-100; 2mM DTT; 100 μg/ml Cycloheximide and 1X Protease-Inhibitor Cocktail EDTA-free (Roche).

**Sucrose gradient fractionation and ribosome profiling :**

For sucrose gradient fractionation of LCLs, 7 A260 units of the cleared cell lysate were incubated with 100U or 500U of RNase I (Ambion) or 5U or 15U of RNase I (Epicentre) or 300 Units of RNase T1 (Fermentas) and 500ng of RNase A (Ambion) for 30 minutes at room temperature. RNase digestion was stopped with 20 mM Ribonucleoside Vanadyl Complex (NEB: S1402S). No RNase control included 20 mM Ribonucleoside Vanadyl Complex in cell lysis buffer to inhibit any endogenous RNase activity. Samples were loaded on top of a 10-50% (Weight/Volume) sucrose gradient (20 mM Tris-HCl pH 7.5; 150 mM NaCl; 5 mM MgCl2; 1 mM DTT; 100 μg.ml^-1^ Cycloheximide) and centrifuged in a SW-41 rotor at 38,000 rpm for 2h30min at 4ºC.

HEK293 cells were treated similarly with the following differences. 5 A260 Units of the cleared cell lysates were incubated with 75 Units of RNase I (Ambion) or 12 Units of RNase I (Epicentre) or 300 Units of RNase T1 (Fermentas) and 500ng of RNase A (Ambion) for 30 minutes at room temperature or 4ºC. After this, RNase digestion was stopped with 50 Units of SUPERase•In and samples were loaded on top of a 10-50% (Weight/Volume) sucrose gradient (20 mM HEPES-KOH pH. 7.4; 5 mM MgCl2; 100 mM KCl; 2 mM DTT; 100 μg.ml^-1^ of Cycloheximide) and centrifuged in a SW-40ti rotor at 35,000 rpm for 2h40min at 4ºC.

For ribosome footprint library preparation using LCLs, 7 A260 Units of the cleared cell lysates were incubated with 300U Units of RNase T1 (Fermentas) and 500ng of RNase A (Ambion) for 30 minutes at room temperature. Instead of a sucrose gradient fractionation, a 34% (Weight/Volume) sucrose cushion was used. The sample was ultracentrifuged in a TLA120.3 rotor at 4ºC for 4 h at 70,000 rpm. The pellet was resuspended in 700μl of Qiazol reagent from miRNeasy kit. RNA was purified using manufacturer’s instructions. Purified RNA was 3’end dephosphorylated using 10U of T4 PNK (NEB) at 37C for 3 hours. Dephosphorylated RNA was heated to 80C for 90 seconds before loading onto a 15% (Weight/Volume) polyacrylamide TBE-Urea gel that was prerun at 180V for 15min. Electrophoresis was carried out for ~1.5hr at 180V. Custom RNA oligos were used to determine the 26-34 nucleotide size range on the gel and was excised on a DarkReader Transilluminator (Clare Chemical Research). Excised gel pieces were placed in GeBAflex electroelution tubes with 8kDA molecular weight cutoff (Gerard Biotech). Samples were electroeluted at 120V for 90min, and the eluate was filtered through Spin-X 0.22um Cellulose Acetate centrifuge filter tubes (Sigma). 300 mM sodium acetate pH 5.5, 10 mM MgCl_2_, and 22.5 μg of GlycoBlue (Life Technologies) was added to filtrate. Equal volume of 100% isopropanol was used for precipitation at -20°C overnight. RNA was pelleted by centrifugation at 21000g at 4C. RNA pellet was washed once with cold 75% ethanol and resuspended in 6ul of nuclease free water. NEBNext Small RNA Library preparation kit was used to convert the isolated RNA fragments into Illumina sequencing library using manufacturer’s instructions except for the choice of 30C for 3hrs for all ligation reactions. Initial experiments were carried out without ribosomal RNA depletion, and the most highly represented rRNA sequences were determined from these experiments. Following biotinylated complementary oligos were custom synthesized (IDT), and were used for rRNA depletion as previously described (Ingolia et al., 2012).

| 5’ ACGGTCCCCCGCGAGGGG 3’ |
| --- |
| 5’ TCCCCGCCCCTTGCCTCTC 3’ |
| 5’ GGGGGGATGCGTGCATTTA 3’ |
| 5’ TTGGTGACTCTAGATAACC 3’ |
| 5’ TCGTGGGGGGCCCAAGTC 3’ |
| 5’ CTGGGTCGGGGTTTCGTACGT 3’ |
| 5’ AGGGGCTCTCGCTTCTGGC 3’ |
| 5’ ACCCCTCCTCCCCGCGCCC 3’ |
| 5’ GGGGCCGGGCCGCCCCTC 3’ |

**Comparison of ribosome profiling data generated using RNase I or RNase A+T1:**

Since the seminal development of ribosome profiling for monitoring genome-wide translation in the yeast Saccharomyces cerevisiae (Ingolia et al., 2009), the approach has been used to study translational control in diverse organisms ranging from bacteria to mammals. A critical step in this method is RNase treatment of cell lysates to digest polyribosomes into well-defined 80S monoribosomes (monosomes) carrying protected mRNA fragments. In the original methodology, Ingolia et al., 2009 carefully optimized this digestion step for yeast extract and found that RNase I (which has the desirable property of cutting after all four nucleotides) was the most appropriate choice. However, a protocol adaptation for mammalian cell lysates did not intensively reexamine RNase digestion conditions (Ingolia et al., 2011, 2012). Furthermore, purification of RNase I digested monosomes by sucrose gradient fractionation was replaced with sedimentation through a sucrose cushion. Although simpler, this modification eliminates a crucial quality control step allowing the user to monitor both the extent of polyribosome digestion into monosomes and the intensity of monosome signal after RNase treatment.

While working to establish ribosome profiling, we observed that RNase I digestion caused extensive ribosomal degradation in lysates from three human cell lines (HEK293, HeLa and LCLs). This resulted in an unexpected shift of RNA signal from fractions corresponding to polyribosomes and monosomes to significantly lighter fractions (Figure 1; Supplemental Figure S1). Similar results were observed across a range of digestion conditions using RNase I from two different vendors, including those previously reported (Ingolia et al., 2011, 2012) and those suggested in the ARTseq kit from Epicentre (catalog number: RPHMR12126) (Figure 1 and Supplemental Figure S1). Across all digestion conditions tested, we observed loss of polyribosome signal but this was not accompanied by a corresponding increase in monosome signal. The only RNase I digestion condition that preserved monosome signal intensity required incubation at 4C instead of room temperature, though the total amount of A254 signal was still less than expected (Supplemental Figure S1). In contrast, we found that treatment with RNases A + T1 (which collectively cut after C, U and G) resulted in complete digestion of polyribosomes into monosomes (Figure 1).

To test whether use of alternate RNases could have an impact on data quality, we generated sequencing libraries using RNase A + T1 digested HEK293 cell lysates and compared our sequencing results to those previously published utilizing RNase I digestion in the same cell line^18^. We found that RNase A + T1 digestion yielded a size distribution of mapped footprint reads similar to that observed with RNase I. Consistent with loss of ribosome integrity with RNase I, however, a much higher fraction of reads in RNase I digested libraries mapped to rRNA than in the RNase A + T1 digested libraries (Supplemental Figure S1B). More importantly, when per transcript ribosome footprint counts were compared to an independently generated quantitative protein mass spectrometry dataset (Geiger et al., 2012) for the same cell line, the RNase A + T1 ribosome profiling data correlated significantly better than RNase I (Supplemental Figure S1C-D; p-value < 0.0001).

Our data indicate that when optimizing the footprinting conditions in a new organism/cell line, stringent quality control steps such as sucrose gradient sedimentation are necessary to ensure that the RNase digestion conditions employed do not substantially disrupt ribosome integrity. Otherwise, the mRNA fragments being sequenced may originate from a distinct subset of ribosomes that were differentially resistant to RNase digestion (e.g., because they contain a distinct set of associated proteins (Komili et al., 2007). Our results suggest that nuclease choice should be considered a key parameter. In particular, RNases A + T1 appear to be more suitable than RNase I for ribosome profiling in several human cell lines including LCLs that were used in the current study.

**Extraction of total RNA and mRNA enrichment for RNA-seq experiments:**

Lymphoblastoid cell lines (LCLs) were grown at a density between 3x10^5^ - 6x10^5^ cells/ml. Total RNA was extracted using TRIzol reagent according to the manufacturer’s instructions (Lifetechnologies), then purified using the Qiagen RNeasy kit (Qiagen, Valencia, CA) and treated with RNase-free DNase (Qiagen, Valencia, CA). RNA integrity was checked with a Bioanalyzer (Agilent, Santa Clara CA) and only samples with an RNA integrity number (RIN) of > 9.5 were subsequently subjected to either ribosomal depletion or poly-A-selection. For ribosomal RNA depletion, 5 μg of purified total RNA was depleted of rRNAs using the Ribo-Zero Magnetic Gold Kit (Human/Mouse/Rat) (Epicentre Biotechnologies, Madison, WI) according to the manufacturer’s instructions. For poly-A selection, 10 μg of purified total RNA were enriched by performing two cycles of selection using the Dynabeads mRNA Purification Kit (Life Technologies).

**Preparation of RNA-seq sequencing library preparation:**

Stranded libraries were prepared following the dUTP protocol (Parkhomchuk et al., 2009). In brief: ~100 ng of either rRNA depleted RNA or poly-A-selected RNA were fragmented with 10 x fragmentation buffer (Life Technologies, #AM8740) for 2 min at 70 °C. First-strand cDNA synthesis was primed with random hexamers. Actinomycin D was added to reduce anti-sense artifacts. For the second-strand synthesis dTTP was replaced with dUTP. The cDNA was end-repaired and phosphorylated with the End-It kit from Epicentre (ER0720). After treatment with Klenow fragment (NEB, Cat# M0212s) and dATP, Illumina TruSeq adapters were ligated to the protruding 3_-‘A’ base (LigaFast, Promega Cat#M8221). After size-selection on an agarose gel the second strand was eliminated by digestion with AmpErase Uracil N-glycosylase (UNG) (Life Technologies, N8080096). Following PCR amplification samples were sequenced on the Illumina Hi-Seq 2000. For each cell line, we generated 2 x 101bp paired end RNA-seq data using two biological replicates of ribosomal RNA depleted and three biological replicates of poly-A-selected RNA.

**Data summary:**

HEK293 cell ribosome profiling sequencing data generated after RNase I digestion was downloaded from (Ingolia et al., 2012) (GEO accession: GSE37744). Only data generated using low magnesium chloride (5mM) was used to ensure comparability. HEK293 cell RNase A + T1 digestion data was downloaded from GEO accession: GSE52447 (Ricci et al., 2013). In addition, we generated 50 ribosome profiling libraries from LCLs of 30 individuals. For the same set of individuals, we generated or used existing RNA-seq data from the following sources.

We downloaded RNA-seq data (raw FASTQ files) for 14 samples from the Geuvadis Consortium (Lappalainen et al., 2013), 20 samples from (Pickrell et al., 2010). We generated 44 poly-A selected or rRNA depleted RNA-seq libraries as described above.

Quantitative mass spectrometry data for HEK cells was available from (Geiger et al., 2012). SILAC based absolute protein level quantification for LCLs (Khan et al., 2013) was used for the analysis presented in Figure 2. All other analyses utilized proteomics data that we previously using the same cell lines and relative protein level quantified by iTRAQ method (Wu et al., 2013). Protein levels for ~6000 genes were measured (Wu et al., 2013). For our analyses, we used the protein level measurements for genes with no missing values.

**Sequence alignment and processing:**

To enable comparable analysis of different high throughput sequencing datasets, we employed a uniform alignment and preprocessing pipeline starting from raw reads in FASTQ format. Where applicable, 3’ adaptors were removed using cutadapt v.1.2.1 (Martin, 2011) with the following parameters: --overlap=2 --minimum-length=21 --quality-cutoff=33. Reads were aligned using Bowtie 2 v.2.0.5 with a sequential strategy (Langmead and Salzberg, 2012). Human rRNA and tRNA sequences were downloaded from UCSC Genome Browser (hg19) repeatmasker track (http://www.repeatmasker.org). All reads mapping to these sequences were filtered out. The remaining reads were aligned using parameters “-L 18 --norc” to APPRIS principal transcripts (release 12) (Rodriguez et al., 2013) from the GENCODE mRNA annotation v.15 (Harrow et al., 2012). This step was followed by alignment to all GENCODE transcripts and finally to the human genome (hg19). This strategy was preferred to avoid any differences in mappability of the exon-exon junction spanning reads due to read length differences between ribosome profiling and RNA-seq libraries. We only retained alignments with a mapping quality greater than two for subsequent analyses. Reads mapping to coding regions, 5’UTRs, and 3’UTRs were counted separately using bedtools (Quinlan and Hall, 2010) and custom scripts. For all transcript level analyses, reads that map only to coding regions were used.

We assessed whether alignment to personal genomes as opposed to reference sequence had an impact on our expression measurements. As previously described (Kasowski et al., 2013), we constructed personal transcriptomes by adding phased SNPs (see below for genotype data and processing). This resulted in two transcriptomes per individual: a maternal and a paternal transcriptome. We then aligned reads to each of these two transcriptomes independently, followed by assignment of each read to the transcriptome where it mapped with higher quality. If a read mapped with equal quality to both transcriptomes, it was labeled as ambiguous, but still kept in the pool of total mapped reads. The source code for alignment to personal genomes is available at <https://github.com/oursu/Personal_genome_mapping>. When comparing the gene level read counts obtained using personal genome alignment and alignment against the reference sequence, we found a very strong correlation (Pearson correlation ~0.99, p < 2.2 x 10^-16^). For simplicity, we used alignments obtained using the reference transcriptome for all analyses.

**Ribosome profiling sample identity verification:**

The cell line identity for all ribosomal profiling libraries were verified by comparing empirically generated genotype calls to the reference genotypes. Specifically, we utilized samtools mpileup utility in combination with bcftools (Li et al., 2009) to generate genotype calls from the ribosomal profiling read alignments. Genotypes that were supported by at least 10 reads were retained using ‘vcfutils.pl varFilter -d10’. Finally, a custom perl script was used to compare the number of perfect matches between empirically called genotypes and the reference genotype that was available from the HapMap and the 1000 Genomes Project (see below).

**Genotype data and processing:**

Genome sequences were obtained from the 1000 Genomes Project pilot 2 trios and Phase1v3 (The 1000 Genomes Project Consortium 2012; The International HapMap 3 Consortium 2010) for 27 of the 30 individuals. The genome sequences of three YRI LCLs (NA19139, NA19193, and NA19201) were not available from the 1000 Genomes Project. For these three cell lines, genotypes were imputed from HapMap release 28 data data (The International HapMap 3 Consortium 2010; The International HapMap Consortium 2007) to the 1000 Genomes Phase1v3 reference panel (The 1000 Genomes Project Consortium 2012) as follows.

HapMap release 28 genotypes for the three YRI cell lines, along with that of their trio members (mother, father, child) were obtained. HapMap marker coordinates were lifted from genome assembly NCBI36 (hg18) to GRCh37 (hg19) using the liftOver tool (Hinrichs et al., 2006). For each HapMap trio, genotypes at markers with Mendelian errors were set to missing. HapMap markers not found in the 1000 Genomes Phase1v3 dataset based on matching position, with discordant alleles, or with extreme allele frequency differences between the HapMap (YRI) and the 1000 Genomes (AFR) data were excluded thereby ensuring the proper alignment of the two datasets. After these quality control steps, HapMap genotypes were available at ~3.5 million markers for the three YRI trios.

HapMap haplotypes were inferred in SHAPEIT v2 (Delaneau et al., 2012, 2013) by leveraging both the trio family information and information from the 1000 Genomes Phase1v3 reference haplotypes. Using IMPUTE v2.3.0 (Howie et al., 2012, 2009), NA19139, NA19193, and NA19201 genotypes were then imputed to the 1000 Genomes Phase1v3 reference panel into the haplotypes inferred previously by SHAPEIT (-use_prephased_g option). For both the pre-phasing in SHAPEIT2 and the imputation in IMPUTE2, the 1000 Genomes reference haplotypes were obtained from the IMPUTE2 website (<https://mathgen.stats.ox.ac.uk/impute/impute_v2.html#reference>, December 9 2013 release for chromosome 1 to 22, and August 26 2012 release for chromosome X).

**Processing variant calls for downstream analyses:**

We included all variant calls provided by both release and pilot datasets without additional score or source filtering. We subsetted all single nucleotide polymorphisms (SNPs) that overlap APPRIS transcripts, same set used in sequence read alignments as described above. We converted genome coordinates to 0-based transcriptome coordinates and retained all phasing information from the VCF files. About ~8% of the variants in the pilot dataset were unphased, and for these variants, we randomly assigned the phase.

**Sequence data normalization and quality control:**

We investigated whether specific transcripts had a skewed representation in RNA-seq libraries due to the mRNA enrichment method of choice (Supplemental Figure S2A-B). We generated RNA-seq libraries using either poly-A selection or ribosomal RNA depletion from a subset of the cell lines used in the study (Supplemental Figure S2B). We calculated the logarithm of the mean read counts among the poly(A) selected and rRNA depleted libraries for each transcript across all samples. We found a high correlation between the two approaches but few clear outliers were observed (Spearman correlation coefficient *ρ* ~ 0.93; p < 2.2 x 10^-16^). For example, replication-dependent histones are not polyadenylated hence poly-A selection based RNA-seq libraries had low read counts for these transcripts (Supplemental Figure S2A-B). We then fitted a linear regression to the logarithm of the mean read count in ribosomal RNA depleted libraries as a function of the logarithm of the mean read count in poly-A selected libraries (Supplemental Figure S2A). We defined outliers as transcripts that had an absolute standardized residual greater than three and removed these transcripts from further analyses (Supplemental Figure S2A). The Spearman correlation coefficient improved to ~0.96 upon removal of these outliers (p < 2.2 x 10^-16^). Given that the ribosome profiling method does not include a similar poly(A) selection step, this quality control step is crucial for proper comparison between RNA expression and ribosome occupancy.

We then inspected the impact of PCR biases on our ribosome profiling data. We calculated the number of unique species mapping to each transcript for each library. Unique species is defined by collapsing all identically mapped ribosome profiling reads into one. We then merged all libraries and calculated the total number of reads and species for each transcript. We found a very high correlation between the total number of reads and the number of unique species (Spearman correlation coefficient rho = 0.99; p < 2.2 x 10^-16^; Supplemental Figure S2C). We fitted a spline that approximated the total read counts from the unique species count (Supplemental Figure S2C). We identified transcripts that were outside the 95% confidence interval of the fitted spline, and found that these outlier transcripts belonged almost exclusively to the most highly expressed transcripts such as ribosomal proteins. Furthermore, we observed increasing variation of ratio between reads to species for transcripts with high read counts. These results strongly suggest that multiple ribosome fragments in addition to PCR artifacts were contributing to difference between the two measures. While, we note that it is impossible to differentiate true multiple reads from PCR artifacts in the current experiment, our results implicated that total read counts was a better metric for quantification of ribosome occupancy of entire transcript. Therefore, all subsequent analyses were carried out using total read counts.

At this point, ~9600 transcripts had a read count per million reads mapped (cpm) (as implemented in the edgeR package (McCarthy et al., 2012) greater than one in at least 40 RNA-seq libraries and 36 ribosome profiling libraries. These transcripts were retained for further analysis. We accounted for different library sizes by calculating normalization factors using trimmed mean of M values (Robinson and Oshlack, 2010), and estimated the mean to variance relationship in the data using the voom method (Law et al., 2014). We explicitly specified the individual identifier to indicate which libraries were replicates from the same individual while applying the voom method. The inverse variance weights obtained from the voom method were used in all analyses where applicable.

To account for variability attributable to laboratory specific differences in RNA expression data, we then applied the ComBat method as implemented in the sva R package (Leek et al., 2012). Specifically, we used the data source as a known confounding variable (Pickrell et al (Pickrell et al., 2010), Geuvadis Consortium (Lappalainen et al., 2013), Snyder poly(A) enrichment, or Snyder rRNA depletion). We adopted the parametric empirical Bayes framework to remove the component of variability explained by this known confounding variable from the RNA expression values.

We also tested surrogate variable analysis (SVA) to detect unmeasured confounding effects (Leek et al., 2012). We used a linear model to regress out the identified surrogate variables and found that the residuals after SVA correction for most datasets were very highly correlated with pre-normalization values (Spearman correlation coefficient > 0.99). For subsequent analyses, broadly consistent results were obtained for SVA-corrected and uncorrected data. For simplicity, we only present and discuss the analyses obtained using the data before the SVA-normalization.

We then explored the relationship between transcript length and normalized expression values. Transcript length correction would have no effect on the vast majority of the analyses presented in this work that compare the expression of a given transcript across individuals. However, for analyses comparing RNA expression, ribosome occupancy and protein levels of different transcripts (as described in Figure 2), length biases could potentially impact the results. While there is a dependence on RNA-seq counts and transcript length, we found low correlation between ribosome occupancy and transcript length. Hence, standard length normalization for ribosome profiling data was not justified. More importantly, the observed higher correlation between ribosome occupancy and protein levels compared to RNA expression and protein levels (Supplemental Figure S2F) was robust to transcript length normalization of RNA expression measurements. For simplicity, we presented results obtained without length normalization.

To assess reproducibility and overall quality of the sequencing data after these preprocessing steps, we used two approaches. First approach was to calculate, the Pearson correlation coefficient of normalized expression values of all genes between all pairs of sequencing libraries of each type (RNA-seq or ribosome profiling). We assigned to each library the median of the correlations to all other libraries which indicates the global similarity of the given library to all other sequencing data. We observed that all libraries except one had a median correlation coefficient between ~0.89 and ~0.95 (Supplemental Figure S2D). The single outlier library (an RNA-seq library from Pickrell et al., 2010) was removed from further analyses (Supplemental Figure S2D). We also noted that among ribosome profiling datasets, the interquartile range (IQR) of the median correlation coefficients was 0.91 to 0.92. For RNA-seq datasets, the corresponding IQR was 0.92 to 0.93 suggesting that overall reproducibility of RNA-seq and ribosome profiling libraries was similar (Supplemental Figure S2D).

As a complementary approach we calculated the Euclidean distance between the normalized expression values for all sequencing library pairs. For each library, we calculated the mean Euclidean distance comparing it to all other replicates from the same individual or to libraries generated from other individuals. Replicate experiments from the same individual were much more closer to each other compared to experiments from different individuals (Supplemental Figure S2E). Importantly, RNA-seq datasets generated from the same cell line irrespective of the data source behaved in this manner (Supplemental Figure S2E). Consistent with ’t Hoen et al., 2013, this result suggests that RNA-seq experiments across laboratories can be combined after appropriate normalization (Supplemental Figure S2E).

**Calculation of translation efficiency:**

When combined with RNA expression measurements, ribosome profiling enables the estimation of translation efficiency by capturing a snapshot of the transcriptome-wide ribosome occupancy. Previous studies used a simple log-ratio between ribosome profiling and RNA-seq read numbers as a measure of “translation efficiency” (Ingolia et al., 2009, 2011). However, as pointed out in Larsson et al., 2010, and Olshen et al., 2013, the log-ratio of two random variables (X,Y) has an expected non-zero correlation to either X or Y. Consequently, transcription level changes along with proportional differences in ribosome occupancy can alter the log-ratio, hence this measure cannot be reliably interpreted as translation efficiency.

To address this problem, we treated ribosome profiling and RNA-seq as two experimental manipulations of the RNA pool of the cell. By using the voom method, we explicitly modeled the mean to variance relationship and utilized inverse variance weights for the subsequent steps. This preprocessing strategy enabled the application of the well-established statistical methods in the limma R package (Smyth, 2005). Specifically, we used a linear model where the normalized expression values are dependent on the treatment (RNA-seq or Ribosome profiling) and the individual identifiers. For individuals where replicate measurements for both ribosome profiling and RNA-seq experiments were available, we defined translation efficiency as follows. We fitted a linear model using the “lmFit” function from the limma package to the observed expression values as a function of the predictor variables encoding the experiment type and the individual identifier. We then defined a contrast matrix to obtain the coefficients associated with the difference in ribosome profiling and RNA-seq treatment factors for each of the individuals. These coefficients were used as the individual specific translation efficiency measures. We took the median of these individual specific estimates to derive the transcript-specific translation efficiency measures.

**Self-Organizing maps for integrative gene expression analysis:**

Self-organizing maps (SOMs) enable clustering and visualizing high dimensional data (Kohonen, 1990; Yin, 2008). SOMs are unsupervised machine learning methods that represent the topological structure of the data in lower dimensions without the need for a labeled training data. We used SOMs to explore the relationship between protein levels and the three expression level measurements: RNA levels, ribosome occupancy and translation efficiency. Each expression measurement was correlated independently to protein levels. However, the interplay between the different expression levels and protein levels for any given transcript cannot be captured by simple correlation analysis.

SOMs rely on a suitable measure of distance between the transcripts for the clustering. To avoid skewing distance calculation due to difference in scale and variance of the expression measurements, expression levels and protein amounts were converted to percentiles using the empirical cumulative distribution function for each level. For RNA expression and ribosome occupancy, we used the median normalized expression value across all libraries and for translation efficiency we used the median translation efficiency of the transcript across all individuals. After conversion to percentiles, we retained genes with all four measurements: RNA expression, ribosome occupancy, translation efficiency and protein levels.

We defined the total number of neurons and the x-y dimensions of the SOM as previously described (Xie et al., 2013). We used a toroid with a hexagonal grid for the map. The learning rate of the SOM was reduced linearly from 0.05 to 0.01 during training, and each data point was presented to the map 100 times. Given that the initial configuration of the map is random, the resulting maps can be different from each other. To find the best possible initial configuration, we repeated the SOM training 500 times with different random number generator seeds. We chose the random number seed that minimized mean distances of the data points to the codebook vectors of the winning units in the SOM. The best SOM according to this criterion was achieved by setting R session random number generator seed to 173. The kohonen R package (Wehrens and Buydens, 2007) was used for training the SOM with custom modifications to the plotting functions.

We then clustered the codebook vectors of the 140 units in the SOM using affinity propagation clustering (Frey and Dueck, 2007) as implemented in the apcluster R package (Bodenhofer et al., 2011). We calculated the input pairwise similarity measures using the negative squared distances. The exemplars for affinity propagation clustering were initialized by setting the *q* parameter to 0.25. All transcripts that belonged to SOM units in a given cluster were used for subsequent gene ontology enrichment analysis as described below.

**Gene set enrichment analysis:**

FuncAssociate 2.0 was used for gene set enrichment analyses (Berriz et al., 2009). The ensembl gene identifiers were used for the enrichment tests. The background gene list was explicitly defined as the set of all genes that could potentially be included in the query set. We defined significant enrichments as GO terms with an odds ratio greater than 2 and adjusted p-value < 0.05. P-value adjustment was carried out using a permutation method to account for the overlap between the GO terms. We calculated the Kappa Similarity Score between all pairs of significantly enriched GO terms. We retained edges between all pairwise GO terms whose Kappa similarity score was greater than 0.1. Enriched GO terms were visualized with Cytoscape (Smoot et al., 2011) using the edge-weighted spring embedded layout.

**Analysis of between individual variation in RNA expression and ribosome occupancy:**

Replicate measurements for RNA-seq and ribosome profiling were used to determine inter-individual variance while controlling for platform specific variance observed between replicates from the same individual. To decompose these two variance components, we used a linear mixed effects model where we treated the individual as a random effect. For each transcript, ribosome occupancy level or RNA expression was modeled using the lmer function from the lme4 R package. As before, we utilized the inverse variance weights obtained from the voom approach and fitted the model using log-likelihood instead of a restricted maximum likelihood approach. We tested the null hypothesis that the variance of the random effect is zero. Rejection of the null hypothesis implied that there was significant inter-individual variance in the expression of the given transcript. Since variance is by definition non-negative, this particular hypothesis test is a non-regular problem occurring at the parameter boundary. To overcome this difficulty, we adopted a simulation-based approach using an exact likelihood ratio test implemented in RLRsim R package (Scheipl et al., 2008). Specifically, we compared the mixed effects model defined above to the simple linear model that does not use the random effect component. We calculated the p-value of the log-likelihood ratio test using the exactLRT function from the RLRsim R package. We carried out 500000 simulations for each test to calculate the p-value and empirically confirmed the stability of the p-values. Multiple-hypothesis correction was applied to RNA expression and ribosome occupancy p-values separately using Holm’s method.

**Cis-QTL identification:**

Association between gene expression and the genotype at each variant position located in the exons of the APPRIS transcripts was tested in the set of 21 unrelated Yoruban individuals using PLINK v1.07 (Purcell et al., 2007). For each transcript, replicate gene expression measurements were averaged for this analysis. The expression values were regressed on variant genotypes assuming an additive genetic model where genotype was coded as 0,1, or 2 copies of the alternate allele and restricting the testing to variants with a minor allele frequency >10% in the 21 unrelated Yoruban individuals.

Within a gene, P-values were corrected for multiple testing using the max(T) permutation procedure implemented in PLINK (--mperm) and 10,000 permutations of the phenotype. The EMP2 p-values obtained by this permutation test were corrected for the number of polymorphisms tested within each gene. The QTL FDR was determined based on the distribution of the most significant multiple testing corrected P-value (EMP2, one per gene) using the QVALUE v1.32.0 software (Storey and Tibshirani, 2003).

**Genetic Determinants of Variability in Ribosome Occupancy:**

**Defining upstream open reading frames**

We first subsetted SNPs in the 5’ UTR and called any possible open reading frames in transcript sequences of all individuals. Because phasing information is critical for calling open reading frames, we restricted our analysis to 27 individuals whose phased genome sequences were available from the 1000 Genomes Project (see **Genotype data and processing)**. We used AUG and CUG as potential start codons, and UAG, UAA and UGA as potential stop codons. We decided to include non-canonical CUG starts based on two lines of evidence. First, CUG initiation has been reported in few well-documented cases such as *FGF2*, *VEGF*, and *MYC* (Hann et al., 1988; Meiron et al., 2001; Vagner et al., 1996). Second, recent studies mapping genome-wide translation initiation sites have suggested that upstream translation initiates frequently from non-AUG codon, most prominently at CUG sites (Ingolia et al., 2011). Finally, we compared the uORFs in each individual against the uORFs found in the reference genome and labeled each uORF as gained or lost compared to the reference. We did not include indel events in our analysis. However, we annotated whether indel events were present in the 5’UTR of each transcript. Indel events were present at a low frequency (2.9%) in the final set of uORF variant containing transcripts. We used VCFtools, BEDTools, liftOver and Biopython library in processing of the genotype data sources and calling variant uORFs (Cock et al., 2009; Danecek et al., 2011; Kent et al., 2002; Quinlan and Hall, 2010).

**Testing the effect of uORF events on ribosome occupancy**

To group individuals by uORF differences on a given transcript, we first determined all possible combinations of uORF gain/loss events. For each set of uORF events, we assigned values of 0, 1, or 2 to each individual depending on how many alleles of that individual match the particular combination. We then tested whether the copy number of the uORF variants affects ribosome occupancy of the main coding region using two approaches. In the first approach, we used linear regression assuming replicate samples of the individuals (from independently derived cell lines) are independent. In the second, more conservative approach, we fitted a linear mixed model assuming the difference in cell lines is an individual-specific random effect, i.e. treating the different cell lines of the same individual as “technical replicates”. This is approach is equivalent to considering replicate experiments as resequencing of the same library twice. Therefore, this approach is likely too conservative as the culture-to-culture variability, and experimental variability associated with generating ribosome profiling libraries contribute a significant component of observed variation in the data. We compared the results from two approaches by their p-value based rank ordering of the tested transcripts. (Supplemental Figure S4E). Taking the top 33 genes (based on 5% FDR for the first method), there was 85% overlap when only 8.5% was expected by chance. 32 out of these 33 genes were significant (p<=0.05) under the conservative mixed-model assumptions.

In either method, we utilized the inverse variance weights obtained from the voom method as weights in fitting the linear model. We corrected the p-values for multiple hypotheses testing using Benjamini-Hochberg method. For the significant transcripts, we repeated the linear regression analysis using normalized RNA expression rather than ribosome occupancy to test the association between RNA abundance and the uORF variant. 16 out of 33 significant uORF events were either not significantly associated with changes to RNA levels (p>0.05) or had oppositely signed regression coefficients between ribosome occupancy and RNA levels. Using only the data from the 21 Yorubans, we also repeated the analysis for all significant transcripts that had a uORF variant in at least three individuals. (Supplemental Figure S4F, Supplemental Table S4). In this framework, we could not test 7 of the 33 significant associations between the uORF variant and ribosome occupancy as the variant segregated predominantly in one population. For these instances, the variant affecting the uORF is only one of the possible explanations for the observed difference in the ribosome occupancy for the tested transcript.

**The effect of Kozak region sequence on translation efficiency:**

We defined the Kozak region as the six nucleotides preceding the start codon and the two nucleotides following the start codon. We extracted the nucleotide sequence of this region from all annotated APPRIS transcripts and built a position weight matrix (PWM), which recapitulated the known Kozak sequence (Figure 5A). We tested whether the nucleotide content of the Kozak sequence affected translation efficiency using the Kruskal-Wallis test. Specifically, we tested whether transcripts split into four categories based on the nucleotide at a given position has the same translation efficiency. We corrected the p-value from this test using Bonferroni correction for the 8 tests (number of positions) that were performed.

**Association between Kozak region genetic variants and ribosome occupancy:**

Next, we collected all SNPs that intersect annotated Kozak regions. We scored the variant and the reference Kozak sequence using the PWM matrix obtained above. We coded each variant by the PWM score change and assumed an additive relationship between different positions in the Kozak region and copy number of the allele. We then tested whether the variants in the Kozak regions affect ribosome occupancy of the main coding region using a linear model (lm function in R; see discussion for uORFs for comparison to a linear mixed effect model). We utilized the inverse variance weights obtained by the voom method as before. For all Kozak variants affecting ribosome occupancy, we conducted the same association test using RNA expression level as the phenotype. As for the uORF analysis, we deemed RNA association to be not significant if the nominal p-value was greater than 0.05, or if the regression coefficient had the opposite sign.

**Luciferase reporter assays:**

To assay translation efficiency, we used of a bicistronic luciferase reporter construct (Jang et al., 2006). This construct has an SV40 promoter that drives the expression of a bicistronic transcript that includes both the firefly and *Renilla* luciferase. While the *Renilla* luciferase translation is cap-dependent, firefly luciferase has an Hepatitis C virus (HCV) internal ribosome entry site (IRES) that enables cap-independent translation.

Gene synthesis was carried out by GenScript and gene segments were cloned right in front of the start codon (ATG) of the *Renilla* luciferase using the CloneEZ system (GenScript). A total 13 constructs were generated for six genes. Four of these genes had Kozak variants and two genes had uORF variants. The sequences that were cloned into the vector were:

| ENST00000366628.4 - REFERENCE  GTCGCGACCCTGGTCCGGACCTGACCTGAATTGCGACCCCAACCTGGACTGCTCCCCTGACCGCAACCCCTACCCCCGCCCACCAGT |
| --- |
| ENST00000366628.4 – Kozak Variant  GTCGCGACCCTGGTCCGGACCTGACCTGAATTGCGACCCCAACCTGGACTGCTCCCCTGACCGCAACCCCTACCCCCGCCCACCAGC |
| ENST00000362031.4 - REFERENCE  CTGGCCGCCGGCTCCCTCCGGCAGCAGGGAG |
| ENST00000362031.4 – Kozak Variant  CTGGCCGCCGGCTCCCTCCGGCAGCGGGGAG |
| ENST00000254759.3 - REFERENCE  AAAAGAGGTGGGATCGTTTGTCGCG |
| ENST00000254759.3 – Kozak Variant  AAAAGAGGTGGGATCGTTTGTCGCT |
| ENST00000263461.5 - REFERENCE  CGCCTAAGCGGCGGAACTCTGGAGAGTGCGGAACCTAGGATCCTAGGAAACTGAAGGCAACGGAACCTGGGGGCGATGGAATCCGGGAGCGGTGGAACCCGGAGCCTGTAGAACTTGAATCTAGAGGAACCTGACAGCGGCGCCGAGGGGGCCACCCGGACTCTGTTTGGAACGGAAGCACAGTGTCCGCCGCTTCCTGGTTGCGGGTCAGCGCCCAGGTCCTGGGCTGGCCGCCGGG |
| ENST00000263461.5 - Kozak Variant(-3)  CGCCTAAGCGGCGGAACTCTGGAGAGTGCGGAACCTAGGATCCTAGGAAACTGAAGGCAACGGAACCTGGGGGCGATGGAATCCGGGAGCGGTGGAACCCGGAGCCTGTAGAACTTGAATCTAGAGGAACCTGACAGCGGCGCCGAGGGGGCCACCCGGACTCTGTTTGGAACGGAAGCACAGTGTCCGCCGCTTCCTGGTTGCGGGTCAGCGCCCAGGTCCTGGGCTGGCCGCCAGG |
| ENST00000263461.5 – Kozak Variant (-6)  CGCCTAAGCGGCGGAACTCTGGAGAGTGCGGAACCTAGGATCCTAGGAAACTGAAGGCAACGGAACCTGGGGGCGATGGAATCCGGGAGCGGTGGAACCCGGAGCCTGTAGAACTTGAATCTAGAGGAACCTGACAGCGGCGCCGAGGGGGCCACCCGGACTCTGTTTGGAACGGAAGCACAGTGTCCGCCGCTTCCTGGTTGCGGGTCAGCGCCCAGGTCCTGGGCTGGCCACCGGG |
| ENST00000443597.2 - REFERENCE  AGTCGCCGCTCTACTCTCAACGGTGGCGAGCTGCAGTTGCCAAGTGCCCCGTCCCCGTTGCCATAACAGCAGTACACAACCCCTCCTTCCCCTCGCCCGCTTGCTAAACAGTCCTTCCCTCTCGGGACCAAGGGCCTCTCCAGAACTGCTTCTGATTGAGCAGAAACAGGGAGGAGGACTGAGCTTATCTGACTCCAGAGCTTTCAGGAGGGAAGAAAG |
| ENST00000443597.2 – uORF variant  AGTCGCCGCTCTACTCTCAACGGTGGCGAGCTGCAGTAGCCAAGTGCCCCGTCCCCGTTGCCATAACAGCAGTACACAACCCCTCCTTCCCCTCGCCCGCTTGCTAAACAGTCCTTCCCTCTCGGGACCAAGGGCCTCTCCAGAACTGCTTCTGATTGAGCAGAAACAGGGAGGAGGACTGAGCTTATCTGACTCCAGAGCTTTCAGGAGGGAAGAAAG |
| ENST00000396679.1- REFERENCE  CGCGCAGAAGTCCGGGGCTGGCAAGCGCTTCCTGCGCAGCGCCTAGGCGACCTGGAGTTTGTGACGCTGTGATGGTCTAGAGGCTGGAGATTCAAGATCTGGGTGCCATCATTTTCTGGTTCTGTTGATGACCCTCTTCCAGGTTACATACAGCTTACATCTTGCATCCTCAAGCGTTTTTCTTATAAGGCTAAAAATTCACAAAGCATATATCA |
| ENST00000396679.1 – uORF variant  CGCGCAGAAGTCCGGGGCTGGCAAGCGCTTCCTGCGCAGCGCCGAGGCGACCTGGAGTTTGTGACGCTGTGATGGTCTAGAGGCTGGAGATTCAAGATCTGGGTGCCATCATTTTCTGGTTCTGTTGATGACCCTCTTCCAGGTTACATACAGCTTACATCTTGCATCCTCAAGCGTTTTTCTTATAAGGCTAAAAATTCACAAAGCATATATCA |

The bicistronic constructs were transfected into HEK293 cells using lipofectamine 2000 (Invitrogen) in a 96 well format following manufacturer’s guidelines. Specifically, 200ng of DNA was mixed with 0.5ul of lipofectamine for each well. The mixture was incubated for 30 min at room temperature to facilitate complex formation. HEK cells were 75-90% confluent at the time of transfection. Cells were washed once with PBS and 100ul of OPTI-MEM was added to all wells. 20ul of the DNA-lipofectamine mixture was added to each well dropwise. Cells were incubated for 4 hrs at 37C. At this point, cells were washed again with PBS and the media is changed to DMEM. The cells were lysed after 24 hours and assayed for luciferase activity using Dual Luciferase Reporter Assay System (Promega). All luciferase experiments were carried out in a blinded experimental setting such that the experimenter did not know the identity of the tested constructs. Cap dependent translation was calculated by taking the ratio of *Renilla* (cap) to firefly (HCV IRES) luciferase activity and derived from 5 replicate experiments for the Kozak variants and 4 replicates for the uORF variants. The HCV-IRES dependent translation of firefly luciferase accounted for differences in RNA expression and transfection efficiency. Outlier detection was carried out as described (Jacobs and Dinman, 2004) which resulted in removal of one data point each for the WDR11 -3 and -6 position variants. The differences between *Renilla* to firefly luciferase ratios were assessed using Welch two sample two sided t-test. Except two Kozak variants, all tested genes had statistically significant differences and were shown in Figure 5 and Supplemental Figure S5.

**Supplemental References:**

[Cock, P.J.A., Antao, T., Chang, J.T., Chapman, B.A., Cox, C.J., Dalke, A., Friedberg, I., Hamelryck, T., Kauff, F., Wilczynski, B., et al. (2009). Biopython: freely available Python tools for computational molecular biology and bioinformatics. Bioinformatics *25*, 1422–1423.](http://paperpile.com/b/hDBL17/EKac)

[Danecek, P., Auton, A., Abecasis, G., Albers, C.A., Banks, E., DePristo, M.A., Handsaker, R.E., Lunter, G., Marth, G.T., Sherry, S.T., et al. (2011). The variant call format and VCFtools. Bioinformatics *27*, 2156–2158.](http://paperpile.com/b/hDBL17/ljou)

[Delaneau, O., Marchini, J., and Zagury, J.-F. (2012). A linear complexity phasing method for thousands of genomes. Nat. Methods *9*, 179–181.](http://paperpile.com/b/hDBL17/ayTV)

[Delaneau, O., Zagury, J.-F., and Marchini, J. (2013). Improved whole-chromosome phasing for disease and population genetic studies. Nat. Methods *10*, 5–6.](http://paperpile.com/b/hDBL17/Ij3e)

[Geiger, T., Wehner, A., Schaab, C., Cox, J., and Mann, M. (2012). Comparative proteomic analysis of eleven common cell lines reveals ubiquitous but varying expression of most proteins. Mol. Cell. Proteomics *11*, M111.014050.](http://paperpile.com/b/hDBL17/RRFh)

[Hinrichs, A.S., Karolchik, D., Baertsch, R., Barber, G.P., Bejerano, G., Clawson, H., Diekhans, M., Furey, T.S., Harte, R.A., Hsu, F., et al. (2006). The UCSC Genome Browser Database: update 2006. Nucleic Acids Res. *34*, D590–8.](http://paperpile.com/b/hDBL17/MzDZ)

[Howie, B., Fuchsberger, C., Stephens, M., Marchini, J., and Abecasis, G.R. (2012). Fast and accurate genotype imputation in genome-wide association studies through pre-phasing. Nat. Genet. *44*, 955–959.](http://paperpile.com/b/hDBL17/VPx5)

[Howie, B.N., Donnelly, P., and Marchini, J. (2009). A flexible and accurate genotype imputation method for the next generation of genome-wide association studies. PLoS Genet. *5*, e1000529.](http://paperpile.com/b/hDBL17/E1r8)

[Kent, W.J., Sugnet, C.W., Furey, T.S., Roskin, K.M., Pringle, T.H., Zahler, A.M., and Haussler, D. (2002). The human genome browser at UCSC. Genome Res. *12*, 996–1006.](http://paperpile.com/b/hDBL17/wNcY)

[Komili, S., Farny, N.G., Roth, F.P., and Silver, P.A. (2007). Functional specificity among ribosomal proteins regulates gene expression. Cell *131*, 557–571.](http://paperpile.com/b/hDBL17/jMl9)

[Larsson, O., Sonenberg, N., and Nadon, R. (2010). Identification of differential translation in genome wide studies. Proc. Natl. Acad. Sci. U. S. A. *107*, 21487–21492.](http://paperpile.com/b/hDBL17/G4XG)

[Martin, M. (2011). Cutadapt removes adapter sequences from high-throughput sequencing reads. EMBnet.journal *17*, 10–12.](http://paperpile.com/b/hDBL17/EhZE)

[Olshen, A.B., Hsieh, A.C., Stumpf, C.R., Olshen, R.A., Ruggero, D., and Taylor, B.S. (2013). Assessing gene-level translational control from ribosome profiling. Bioinformatics](http://paperpile.com/b/hDBL17/fucg) *[29](http://paperpile.com/b/hDBL17/fucg)*[, 2995–3002.](http://paperpile.com/b/hDBL17/fucg)

[Quinlan, A.R., and Hall, I.M. (2010). BEDTools: a flexible suite of utilities for comparing genomic features. Bioinformatics *26*, 841–842.](http://paperpile.com/b/hDBL17/3iy2)

[Storey, J.D., and Tibshirani, R. (2003). Statistical significance for genomewide studies. Proc. Natl. Acad. Sci. U. S. A. *100*, 9440–9445.](http://paperpile.com/b/hDBL17/KsdV)

Yin, H. *The Self-Organizing Maps: Background, Theories, Extensions and Applications* 715-762 (Springer Berlin Heidelberg, 2008).
