# Supplementary material for: Integrative analysis of RNA, translation, and protein levels reveals distinct regulatory variation across humans
Source: Genome Res. 2015 Nov;25(11):1610–21. doi: 10.1101/gr.193342.115 (PMC4617958; doi:10.1101/gr.193342.115)
Supplement: Supplemental Material [file supp_gr.193342.115_Supplemental_Figure_and_Table_Legends.docx]

**Supplemental Figure and Table Legends:**

**Supplemental Figure S1. Comparison of RNase I to RNase A + T1 for ribosome profiling**

**(A)** HEK293 cells were pretreated with cycloheximide for 10 minutes before cell lysis. 5 A260 Units of the cleared cell lysates were incubated with 75 Units of RNase I (Ambion) or 12 Units of RNase I (Epicentre) or 300 Units of RNase T1 (Fermentas) and 500ng of RNase A (Ambion) for 30 minutes at room temperature or 4°C. After this, RNase digestion was stopped with 50 Units of SUPERaseIn and samples were loaded on top of a 10-50% (Weight/Volume) sucrose gradient and ultracentrifuged at 4°C. **(B)** Ribosome footprinting data for RNase A + T1 treated HEK293 (Ricci et al., 2014) was compared to data using RNase I digestion (Ingolia et al., 2012). Sequencing reads were aligned sequentially as described in methods. The fraction of reads mapping to rRNA, pre-rRNA, transcriptome, other genomic regions, and unaligned reads were plotted. **(C)** Quantitative mass spectrometry data from HEK293 cells was retrieved from Geiger et al., 2012. Transcripts with at least 10 reads in both RNase I and RNase A+T1 ribosome profiling data were retained. We required that at least one replicate of the mass spectrometry data to have a non-missing value. When multiple replicates were available for mass spectrometry data, we used the mean iBAQ value across replicates. The correlation between the mean iBAQ value and the number of ribosome profiling reads per transcript (log scale) was 0.66 for RNase A + T1 library compared to 0.62 for the RNase I library (Pearson correlation coefficient; p-val < 2.2 x 10^-16^). **(D)** The statistical significance of the difference in correlation coefficients between RNase A + T1 and RNase I library was assessed using a permutation test (p-value < 0.0001). Specifically, we swapped the transcript read counts with 0.5 probability between RNase I and RNase A + T1 datasets. We then calculated the correlation coefficient between the permuted expression values and protein levels. We carried out 10000 permutations and plotted the distribution of absolute differences in correlation coefficients.

**Supplemental Figure S2. Assessing the overall reproducibility and quality of sequencing data**

**(A)** To enrich mRNAs, RNA-seq protocols use poly(A) selection or ribosomal RNA (rRNA) depletion. To identify biases attributable to mRNA enrichment method, we generated RNA-seq libraries using both approaches. We generated 18 RNA-seq libraries using poly(A) selection and 26 RNA-seq libraries using rRNA depletion. For each transcript, we calculated the mean number of mapped reads among all poly(A) selected or rRNA depleted libraries. As expected, the mean read counts were highly similar regardless of mRNA enrichment strategy for the vast majority of the transcripts. We calculated the standardized residuals for each transcript using a linear regression fit (blue line; top panel). Red color indicates transcripts that had absolute standardized residuals greater than three (bottom panel). As expected, histone genes were significantly under-represented in poly(A) selected libraries compared to rRNA depleted libraries. All genes shown in red were removed from further analyses. **(B)** Each cell line is plotted separately to highlight robustness of the comparison between poly(A) selected and rRNA depleted libraries. **(C)** To explore the impact of PCR artifacts on our ribosome occupancy data, we compared the number of mapped reads to species counts, which is obtained by collapsing all identical ribosome footprints into a one. We calculated species counts and read counts for each individual dataset and summed the counts across all datasets. We observed a higher variance in the ratio of these quantities for the transcripts with the highest read counts. Furthermore, transcripts with the largest difference between read and species counts corresponded to transcripts with the highest expression levels including ribosomal proteins. **(D)** We calculated the Pearson correlation coefficient of normalized expression values between all pairs of ribosome profiling libraries. For each library, the median of the correlations to all other libraries was plotted. The interquartile range (IQR) of the median correlation coefficients for ribosome profiling libraries was 0.91 to 0.92. A single RNA-seq library which had a median correlation coefficient ~0.85 was removed from further analysis. The IQR for all RNA-seq libraries was 0.92 to 0.93. **(E)** For all sequencing libraries, the mean pairwise Euclidean distance to replicates from the same individual (grey bars) or to all other individuals (blue bars) was calculated using normalized expression values across all transcripts. Regardless of RNA-seq data source, replicates were more similar to each other than to other individuals. **(F)** We quantified RNA expression and ribosome occupancy as the median expression value across all the datasets (Spearman correlation coefficient *ρ* was shown; p < 2.2 x 10^-16^ for all comparisons). Ribosome occupancy correlated better with protein levels than RNA expression with protein levels (Permutation test, p-value < 0.0001). We also calculated outlier robust correlations (cor_R_) using the Donoho-Stahel projection based estimator as implemented in the R package *robust* **(G)** 10000 permutations were used to assess the significance of the difference in correlation coefficients between ribosome occupancy and protein levels vs. RNA expression and protein levels. The distribution of absolute differences in correlation coefficients in the permuted data and the observed difference was shown. **(H)** The number of genes in each neuron was plotted for the trained SOM in Figure 2. The range of genes per neuron was 4-42 and the interquartile range was 19 - 28.

**Supplemental Figure S3. Comparing individual differences in protein levels, RNA expression and ribosome occupancy**

**(A)** Ribosome occupancy and RNA expression was modeled using a linear mixed model treating individuals as a random effect and mean expression as the fixed effect. A simulation based exact likelihood ratio test (Scheipl et al., 2008) was used to compare the linear mixed model to a linear model that did not include the individual as a predictor. Genes that show significant inter-individual in RNA expression or inter-individual variation in ribosome occupancy were identified (Holm’s corrected p-val < 0.05). We tested whether different sensitivities between the two expression measurements were responsible for the observed difference in Figure 3A-B. We calculated the likelihood ratio test (LRT) statistic for each gene and plotted the fraction of genes that were deemed significant at a given likelihood ratio test statistic. We observed that for both methods all genes with LRT statistic greater than 27 were deemed significant. We restricted the analysis to genes that had LRT statistics greater than 27, and depicted the overlap between genes that show significant inter-individual in RNA expression or inter-individual variation in ribosome occupancy using a Venn diagram. This result suggests the robustness of the conclusion presented in Figure 3A-B to potential differences in sensitivities of the two expression measurements. **(B)** Median log_2_ –counts per million of all RNA expression libraries were compared to that of ribosome occupancy libraries. Presence (black) or absence (blue) of protein level measurements (Wu et al. 2013) indicated that our protein measurements were skewed towards more highly expressed transcripts. **(C)** For each transcript, Spearman correlation coefficients were calculated and plotted between individual specific ribosome occupancy and either protein levels or RNA expression. Genes with no significant inter-individual variation in RNA expression or ribosome occupancy were depicted with white bars. Blue bars correspond to genes that showed significant inter-individual variation in both RNA expression and ribosome occupancy. **(D)** Spearman correlation coefficient distributions were plotted as in panel (a) for genes that exhibit significant inter-individual variation only in RNA expression but not ribosome occupancy. **(E)** We calculated the inter-individual weighted coefficient of variation (CV) for RNA expression and ribosome occupancy. For all genes that exhibit significant variability in both RNA expression and ribosome occupancy, we calculated the ratio of their respective inter-individual CVs. We separated these genes into three classes based on this ratio: higher variability in RNA expression (25% higher CV in RNA expression), higher variability in ribosome occupancy (25% lower CV in RNA expression) and equal variability.

**Supplemental Figure S4. Ribosome occupancy quantitative trait loci (roQTLs) and the effect of nucleotide variants modifying upstream open reading frames on ribosome occupancy**

**(A)** We carried out QTL mapping approach using the unrelated Yoruban individuals in our dataset to identify cis-associations between single nucleotide polymorphisms (SNPs) and ribosome occupancy. We identified cis-roQTLs for 67 genes (30% FDR). For these roQTLs, corresponding nominal p-values corresponding to association with RNA expression were calculated using the same set of individuals. For 34 roQTLs, the nominal p-value of RNA expression association was greater than 0.05. **(B)** For 16 out of 67 genes with roQTLs, we had relative protein level measurements from the same individuals from (Wu et al., 2013). The distribution of nominal p-values for association to protein levels was shown as in panel (a). **(C)** For all significant roQTLs, the effect size of association was determined as the change in expression per alternative allele. The relationship between the effect sizes on ribosome occupancy and RNA expression was shown. Red color indicates that the identified roQTL has a nominal p-value < 0.05 for association with RNA expression. **(D)** Effect size of association was compared as in (c) for ribosome occupancy and relative protein levels. **(E)** We tested the effect of nucleotide variants altering uORFs using two approaches. In the first approach, we used a linear regression that segregated all datasets into three classes solely based on the number of uORF events that differ from the reference sequence. We alternatively reasoned that replicates could be modeled as an individual-specific random effect in a linear mixed-model regression. To compare whether the two approaches gave similar results, we plotted the percentage of overlap between the lists of significant genes from the two models. For example, taking the top 50 significant genes from each list gives 85% overlap with each other when only 8.5% overlap is expected by chance. The consistently high percentage of overlap observed across all values of N indicates rank-order consistency between the results of the two approaches. Furthermore, 32 out of 33 transcripts that test significant (FDR<=0.05) under the linear regression model are also significant (p<=0.05) under the mixed-model assumptions. **(F)** We inspected whether population stratification could explain the significant differences in ribosome occupancy associated with uORF events. We first plotted the matrix of alternate uORF alleles across all individuals as a heatmap. Individuals were color-coded by population group. Light green indicates heterozygotes and dark green depicts individuals homozygous for the alternate allele. Rows represent the 33 uORF variant associated transcripts with significant association with ribosome occupancy (FDR<=0.05). Indicated in red are the 7 transcripts that have less than three Yoruban individuals (out of the 21 total) sharing the minor uORF variant. For the 26 remaining transcripts that have at least three Yoruban individuals with alternate uORF alleles, we performed association analysis using only the data from Yoruban individuals. 21 out of these 26 transcripts were significant (p<=0.05; Supplemental Table S5). **(G)** We examined whether uORF presence correlates with an increase or decrease in ribosome occupancy of the main coding region. For transcripts that contain only one variant uORF and are significantly associated with changes in ribosome occupancy (FDR<=0.2), the distance between the start codon of the uORF and the start codon of the main coding ORF is plotted against the effect size from the linear regression model. Color of the dot represents whether the uORF has been gained or lost compared to the reference sequence. There is no notable bias towards positive or negative direction of change in ribosome occupancy. Furthermore, no correlation is observed between the direction of change in ribosome occupancy and the distance of the uORF to the main start codon.

**Supplemental Figure S5. Kozak sequence impacts translation efficiency and variants modifying Kozak sequence alter ribosome occupancy but not RNA expression**

**(A)** At each position within the Kozak region, the effect of different nucleotides on translation efficiency was shown. All transcripts are split into four categories based on the nucleotide content at the given position of the Kozak region. The boxplots depict the inter-quartile range of translation efficiencies for transcripts with the given nucleotide at the given position. The thick horizontal bar reflects the median of the distribution. To emphasize the differences in median translation efficiency, transcripts with translation efficiencies outside the inter-quartile range were not shown. **(B)** We identified the change in the PWM score for each analyzed Kozak variant. A negative score indicates that tested variant reduces the PWM score. **(C)** Variants in the Kozak region for SNX6, METTL21A, FAM188A were significantly associated with the ribosome occupancy of the coding region. Interestingly, the same variant either did not have a significant association to RNA expression or was associated with a change in the opposite direction (METTL21A). To test the robustness of the associations to statistical modeling assumptions, we also used a more conservative linear mixed modeling approach and denoted the corresponding p-values with p_LME_ [(](https://paperpile.com/c/hDBL17/ET0s)see Methods[)](https://paperpile.com/c/hDBL17/ET0s). Boxplots were used to visualize the distribution of ribosome occupancy and RNA expression for individuals with the given number of non-reference alleles. The horizontal bar reflects the median of the distribution and the box is drawn to depict the inter-quartile range. **(D)** 5’UTRs with or without uORF variants were cloned into a translation efficiency reporter. The reporter expresses a biscistronic mRNA where the *Renilla* luciferase is translated under the control of the cloned 5’UTR and the Firefly luciferase is translated under the control of Hepatitis C virus (HCV) internal ribosome entry site (IRES). The ratio of *Renilla* to Firefly luciferase activity was plotted for LRRC23 and CENPK. Error bars represent s.e.m. The difference between the ratios was assessed using a two sided two sample t-test (* denotes p-value < 0.05).

**Supplemental Table S1. Complete list of Gene Ontology (GO) term enrichments for the clusters identified in Figure 2**

FuncAssociate (Berriz et al., 2009) was used to determine significantly enriched gene ontology (GO) terms. Given the dependencies between GO terms, a permutation based multiple hypothesis testing correction was adopted (Berriz et al., 2009). ENSEMBL Gene identifiers were used for all analyses. Significant GO term enrichments were found for four of the nine clusters shown in Figure 2.

**Supplemental Table S2. Complete list of Gene Ontology (GO) term enrichments for genes exhibit significant inter-individual variability in ribosome occupancy, and RNA expression, related to Figure 3**

GO term enrichment was carried out as described in Supplemental Table S1. The background gene list was explicitly defined as all tested genes. Three sets were used based on variability in expression between individuals: (1) genes with significant variation in both ribosome occupancy and RNA expression (sheet name: Joint) (2) genes with significant variation in only RNA expression (sheet name: RNAExpression) (3) genes with significant variation in only ribosome occupancy (sheet name: RibosomeOccupancy). We used the ‘ordered’ mode in FuncAssociate for the analysis of genes in categories (2) and (3). The likelihood ratio statistic obtained from the simulation based exact likelihood ratio test was used to rank genes (Methods). In ordered mode, FuncAssociate identifies the top *M* genes with the highest likelihood ratio that gives the smallest p-value of enrichment. This procedure was previously described (Berriz et al., 2003).

**Supplemental Table S3. List of genes that exhibit significant variability in ribosome occupancy between individuals but no apparent variability at the RNA-level, related to Figure 3**

**Supplemental Table S4. List of ribosome occupancy cis-QTLs**

Variants with a minor allele frequency >10% in the 21 unrelated Yoruban individuals were selected. For each transcript, replicate gene expression measurements were averaged. Expression values were regressed on variant genotypes assuming an additive genetic model. P-values were corrected for multiple testing using the max(T) permutation procedure implemented in PLINK (--mperm) and 10,000 permutations of the phenotype. The EMP2 p-values obtained by this permutation test were corrected for the number of polymorphisms tested within each gene.

**Supplemental Table S5. Association between ribosome occupancy and genetic variants that modulate uORFs, related to Figure 4**

Summary statistics regarding the number of uORFs and genetic variants that modulate uORF status are presented in “uORF_summary”. The results of association tests between uORF changes and ribosome occupancy of the main ORF are presented in “Association_results”.

**Supplemental Table S6. List of common human polymorphisms (dbSNP release 142) that affect Kozak region of APPRIS transcripts, related to Figure 5**
